# Supplementary material for: Stratified medicine in European Medicines Agency licensing: a systematic review of predictive biomarkers
Source: BMJ Open. 2014 Jan 25;4(1):e004188. doi: 10.1136/bmjopen-2013-004188 (PMC3913033; doi:10.1136/bmjopen-2013-004188)
Supplement: Author's manuscript [file bmjopen-2013-004188.draft_revisions.pdf]

# Stratified medicine in European Medicines Agency licensing: a systematic review of predictive biomarkers

| | |
|---|---|
| Journal: | *BMJ Open* |
| Manuscript ID: | bmjopen-2013-004188 |
| Article Type: | Research |
| Date Submitted by the Author: | 07-Oct-2013 |
| Complete List of Authors: | Malottki, Kinga; University of Birmingham , Public Health, Epidemiology and Biostatistics<br>Biswas, Mousumi; University of Bristol, School of Social and Community Medicine<br>Deeks, Jon; University of Birmingham, Public Health Epidemiology and Biostatistics<br>Riley, Richard; University of Birmingham, Public Health Epidemiology and Biostatistic<br>Craddock, Charles; Queen Elizabeth Hospital, Centre for Clinical Haematology<br>Johnson, Philip; University of Liverpool, Clatterbridge Cancer Centre NHS Foundation Trust<br>Billingham, Lucinda; University of Birmingham, Public Health Epidemiology and Biostatistics |
| <b>Primary Subject Heading</b>: | Pharmacology and therapeutics |
| Secondary Subject Heading: | Public health |
| Keywords: | THERAPEUTICS, Molecular aspects < ONCOLOGY, Molecular diagnostics < INFECTIOUS DISEASES |
| | |

SCHOLARONE™ Manuscripts

For peer review only - http://bmjopen.bmj.com/site/about/guidelines.xhtml

# Stratified medicine in European Medicines Agency licensing: a systematic review of predictive biomarkers

Kinga Malottki*, Mousumi Biswas, Jonathan J. Deeks, Richard D. Riley, Charles Craddock, Philip Johnson, Lucinda Billingham
* corresponding author

Kinga Malottki
Research Fellow
MRC Midland Hub for Trials Methodology Research, University of Birmingham, Birmingham, B15 2TT, UK
k.malottki@bham.ac.uk

Mousumi Biswas
Research Associate
The Discovery Research Programme, School of Social and Community Medicine, University of Bristol, Bristol, BS8 2PS, UK

Jonathan J. Deeks
Professor
Public Health, Epidemiology and Biostatistics, School of Health and Population Sciences, University of Birmingham, Birmingham, B15 2TT, UK
MRC Midland Hub for Trials Methodology Research, University of Birmingham, Birmingham, B15 2TT, UK

Richard D. Riley
Reader
Public Health, Epidemiology and Biostatistics, School of Health and Population Sciences, University of Birmingham, Birmingham, B15 2TT, UK
MRC Midland Hub for Trials Methodology Research, University of Birmingham, Birmingham, B15 2TT, UK

Charles Craddock
Professor
Centre for Clinical Haematology, Queen Elizabeth Hospital, Birmingham, B15 2TH, UK

Philip Johnson
Professor
University of Liverpool & Clatterbridge Cancer Centre NHS Foundation Trust, Liverpool L69 3GA, UK

Lucinda Billingham
Professor
MRC Midland Hub for Trials Methodology Research, University of Birmingham, Birmingham, B15 2TT, UK
Cancer Research UK Clinical Trials Unit, University of Birmingham, Birmingham, B15 2TT, UK

For peer review only

**Abstract**

**Objectives** Stratified medicine is often heralded as the future of clinical practice. Key part of stratified medicine is the use of predictive biomarkers, which identify patient subgroups most likely to benefit (or least likely to experience harm) from an intervention. We investigated how many and what predictive biomarkers are currently included in European Medicines Agency licensing.

**Methods and eligibility criteria** Indications and contraindications of all drugs considered by the EMA and published on their website were screened to identify predictive biomarkers. For all included Biomarker-Indication-Drug (B-I-D) combinations data was collected on: the type of the biomarker, whether it selected a subgroup of patients based on efficacy or toxicity, therapeutic area, marketing status, date of licensing decision, date of inclusion of the biomarker in the indication or contraindication, and on orphan designation.

**Results** 49 B-I-D combinations were identified over 16 years, which included 37 biomarkers and 41 different drugs. All identified biomarkers were molecular. Six drugs (relating to 10 B-I-D combinations) had an orphan designation at the time of licensing. The identified B-I-D combinations were mainly used in cancer and HIV treatment, but also in hepatitis C and three other indications (cystic fibrosis, hyperlipoproteinemia type I, and methemoglobinemia). In 45 B-I-D combinations biomarkers were used as predictive of drug efficacy and in four of drug toxicity. It appeared that there was an increase in the number of B-I-D combinations introduced each year, however the numbers were too small to identify any trends.

**Conclusions** Given the large body of literature documenting research into potential predictive biomarkers and extensive investment into stratified medicine, we identified relatively few predictive biomarkers included in licensing. These were also limited to a small number of clinical areas. This might suggest a need for improvement in methods of translation from laboratory findings to clinical practice.

For peer review only

## Article summary

### Article focus

- To identify predictive biomarkers included in European Medicines Agency licensing
- For identified biomarkers, to investigate their type, the clinical areas in which these biomarkers are used and possible trends over time with regard to the number of new predictive biomarkers considered each year

### Key messages

- 49 Biomarker-Indication-Drug (B-I-D) combinations were identified over 16 years, which included 37 biomarkers and 41 different drugs. There appeared to be an increase in the number of B-I-D combinations introduced each year, however the numbers were too small to identify any trends.
- All identified biomarkers were molecular. They were mainly used in cancer and HIV treatment, but also in hepatitis C and three other indications (cystic fibrosis, hyperlipoproteinemia type I, and methemoglobinemia).

### Strengths and limitations of this study

- Our research, to our knowledge, provides the first indication of the number and nature of predictive biomarkers included in licensing in Europe using systematic review methodology.
- It is likely that the 49 identified B-I-D combinations do not represent a complete list of predictive biomarkers used in practice, as some could have been considered by national regulatory agencies, particularly for drugs considered before EMA was established in 1995.

For peer review only

## Introduction

Drugs are rarely effective in all patients and may be associated with serious adverse events.[1] The challenge of stratified medicine is to identify predictive biomarkers that identify patient subgroups (or strata) with a differential therapeutic response to a linked intervention, allowing more appropriate and effective use of interventions to maximise patient benefit and minimise the occurrence of serious adverse events.[2;3] Predictive biomarkers are defined particular to a treatment for a condition, where biomarker values are associated with differential efficacy or toxicity of that treatment.[4-7] The use of predictive biomarkers promises a more appropriate choice of treatment: it can also help to rationalise funding decisions, avoiding costs of futile treatment and of adverse events. However the additional cost of measuring the marker has to be taken into account. Examples of predictive biomarkers include tamoxifen use in breast cancer, which is prescribed to women who are oestrogen receptor positive,[8] and trastuzumab which is prescribed to those with HER2 overexpression in their tumour.[9]

There is a large body of literature documenting research into potential predictive biomarkers,[10;11] and millions of pounds have been invested into stratified medicine, both in industry and through programs from funding bodies such as the Medical Research Council[12] and Cancer Research UK.[13] We aimed to evaluate the degree to which this investment has led to production of biomarker-treatment combinations ready for use in clinical practice. To explore this question, we have undertaken a systematic review of predictive biomarkers reported in licensing decisions of the European Medicines Agency (EMA).

In our review we aimed to find out how many of the indications and contraindications considered by the EMA define a patient population using a predictive biomarker. We were also interested in the disease areas where predictive biomarkers have been used and any trend over time. It has been hypothesised that stratified medicine has not been implemented in practice as much as expected. This paper provides evidence of the impact of stratified medicine research to date and if less than expected, then this will highlight the need to review the underlying reasons and address the problems.

## Methods

We defined a Biomarker-Indication-Drug (B-I-D) combination as the unit of our analysis, relating to the use of a predictive biomarker with a particular drug for a particular condition or disease.[4-7] For toxicity biomarkers where the a biomarkers of drug toxicity may be used in more than one disease area we grouped these into one B-I-D combination.

All drugs listed on the EMA website in either European Public Assessment Reports or Pending Decisions[14;15] (accessed on the 17th of January 2013) were evaluated, together with their indications and contraindications.

Our inclusion criteria were that the biomarker had to:

For peer review only

(i) be used in the indication and/or contraindication of the drug,
(ii) be associated with a particular treatment,
(iii) identify a subgroup of patients with a particular disease eligible for treatment with the drug.

We excluded biomarkers:

(i) associated with a non-therapeutic substance (for example vaccines),
(ii) not used as predictive, including:
- used for diagnosis, screening or forming part of the disease definition (already established for defining a disease) or established disease subtype,
- prognostic only (associated with outcome regardless of treatment and not predictive of treatment response[16])

(iii) associated with another treatment (for example the biomarker was not associated with the differential efficacy or toxicity of the drug of interest, but another drug given in combination with the drug of interest).

We have reviewed EMA licensing, as in Europe a centralised drug evaluation by the EMA is required for drugs for treatment of a number of conditions, drugs obtained from biotechnology processes and all drugs used for rare conditions (orphan medicines). Companies can also apply for a centralised marketing authorisation of other drugs.[17] Although the EMA does not license biomarkers, it evaluates drugs in groups defined by predictive biomarkers (for example trastuzumab is licensed for use in HER2 overexpressing breast cancer patients).[18] Our approach is likely to give a broad overview of the impact of predictive biomarkers on treatment selection since 1995 (when EMA was established[19]).

In the first stage of screening all entries were screened by two independent reviewers (MB and KM) to identify those potentially including a predictive biomarker. If an entry was identified by at least one of the reviewers as potentially relevant, it was included in the second stage of screening.

In the second stage of screening, a list of potential B-I-D combinations was created based on the entries identified in the first stage. The list of potential B-I-D combinations was assessed by two independent reviewers (MB and KM) using full inclusion/exclusion criteria, based on the information in the Summary of Product Characteristics (which sets out the position of the drug obtained in the assessment process and summarises its properties and clinical use together with the clinical trial evidence that was considered by the EMA)[20] , the Scientific Discussion (which discusses the properties and clinical evidence in more detail) and additional information from targeted internet searches and expert advice if necessary. Any disagreements were resolved by discussion.

For the included B-I-D combinations data was collected on: the type of the biomarker used as predictive, whether it selected a subgroup of patients based on efficacy or toxicity, therapeutic area, marketing status, date of licensing decision, date of inclusion of the biomarker in the indication or contraindication, and on orphan designation (granted to drugs intended for the treatment of a life-threatening or chronically

For peer review only - http://bmjopen.bmj.com/site/about/guidelines.xhtml

debilitating condition which is either affecting no more than 5 in 10,000 people in the EU or when the revenue is unlikely to cover the investment in drug development[21]). To provide a context for our review, we have also collected data on the total number of drugs licensed each year with and without an orphan designation.

**Results**

Across the 18 year period (1995-2012) we identified 49 B-I-D combinations, including 37 biomarkers and 41 different drugs. The details of the review process are presented in Figure 1. Most of the drugs were authorised, the exceptions being:

- Gemtuzumab ozogamicin (refused)
- Zeldoronic acid (pending)
- Imatinib in the indication for aggressive systemic mastocytosis (withdrawn)
- Amprnavir (withdrawn)
- Nelfinavir (withdrawn)

The number of new B-I-D combinations considered by the EMA each year has increased from zero or one per year in the late nineties, to a maximum of 7 in each of 2011 and 2012 as shown in Figure 2. A predictive biomarker was included in the indication or contraindication at the time when the drug was first licensed for 35 drugs (for one (capecitabine) the date of inclusion of the biomarker was unclear from the documentation, for the remaining drugs the time from the initial licensing decision to the inclusion of a predictive biomarker ranged from one to ten years). The proportion of first licensing decision of all new drugs that included a predictive biomarker increased over time and was close to 10% in 2003, 2004, 2011 and 2012 (Figure 3).

Six drugs associated with a predictive biomarker had an orphan designation at the time of licensing, however for two it was removed at the end of exclusivity period (details reported in

For peer review only

Table 1). One of the six drugs (imatinib) was associated with five different predictive biomarkers in five different indications (Figure 4).

The identified predictive biomarkers were all molecular. Thirty-three biomarkers were used to predict treatment efficacy (details reported in

For peer review only

Table 1) and only four to predict toxicity (Table 2).

Most of the biomarkers were included in indications and contraindications of cancer treatments (26 B-I-D combinations) and viral diseases, mainly HIV (17 B-I-D combinations). The remaining biomarkers were used to stratify metabolic and blood disorders (cystic fibrosis, hyperlipoproteinemia type I, and methemoglobinemia) and appeared in the last two years (Figure 2).

## Discussion

Stratified medicine is promoted as key to the future of medicine, and is currently one of the most active areas of clinical research. To our knowledge this review provides the first indication of the number and nature of predictive biomarkers included in licensing in Europe based on the drug indications and contraindications on the EMA website. Forty nine B-I-D combinations were identified. All identified biomarkers were molecular. The identified B-I-D combinations were mainly used in cancer and HIV treatment, with only five used in other disease areas.

It is likely that the 49 identified B-I-D combinations from the EMA database do not represent a complete list of the predictive biomarkers used in practice (some predictive biomarkers could have been considered by national regulatory agencies, particularly for drugs considered before EMA was established in 1995) few are likely to have been omitted, particularly from recent years.[19] Several types of biomarkers were excluded. We did not include biomarkers used for dose adjustments as they do not directly predict efficacy or toxicity (although inappropriate dose adjustment could limit the treatment efficacy or cause adverse events).[22] We also only investigated biomarkers associated with drug treatments. Other biomarkers may be used in practice with non-drug treatments (for example radiotherapy).

The definition of a predictive biomarker can be difficult to apply, as over time predictive biomarkers may become part of a redefinition of the disease or subtype of disease[23] and be classed as diagnostic tests. In our evaluation we excluded diagnostic biomarkers (for example these included factor IX deficiency, or genetic testing for familial lipoprotein lipase deficiency), and biomarkers used to identify an established subtype of a disease (mainly ST segment elevation and non-ST segment elevation myocardial infarction).

The spectrum of diseases where predictive biomarkers have been successfully developed is relatively narrow. This suggests a possible need for more research in other clinical areas. Also the vast majority of the B-I-D combinations were associated with treatment efficacy and only four with toxicity. As adverse events associated with some treatments could be potentially serious and the possibility to screen out patients at high risk prior to commencing treatment would be beneficial. A proportion of the drugs with an associated predictive biomarker identified in our review had an orphan designation. This seems surprising, as convincing evidence to support the use of a drug in a subgroup of patients with a rare

For peer review only

condition might be difficult to obtain, due to the small numbers of patients available to test the hypotheses.

Our review shows that few predictive biomarkers have been included in licensing relative to the large body of literature documenting numerous potential predictive biomarkers.[10;11] Therefore, in spite of the substantial investment in research, the promise of stratified medicine is not yet being realised to a large extent. The reasons for this might include poor translation of findings of laboratory studies into clinical context, or the failure to identify effective predictive biomarkers and treatments. Even though it is becoming easier and cheaper to gather huge sets of genomic data, its interpretation is challenging, which can potentially hinder translational research. Recognising this, initiatives have been undertaken both in the USA (National Institutes of Health and the FDA) and UK (Medical Research Council) to promote the translation of basic research into clinical practice.[12] Also the availability of datasets such as the Cancer Cell Line Encyclopaedia and a similar UK initiative might contribute to the faster progress of stratified medicine.[24;25] The relatively small number of predictive biomarkers identified in licensing might also indicate the need for more sound methodological standards for biomarker discovery and development.[26]

For peer review only

## References

(1) Xinghua Hu S, Foster T, Kieffaber A. Pharmacogenomics and personalized medicine: mapping future value creation. BioTechniques 2005; 39(4).

(2) Aroon DH, Danielle AvdW, Richard DR, Keith A, Karel GMM, Ewout WS et al. Prognosis research strategy (PROGRESS) 4: Stratified medicine research. BMJ 2013; 346.

(3) Trusheim MR, Berndt ER, Douglas FL. Stratified medicine: strategic and economic implications of combining drugs and clinical biomarkers. Nat Rev Drug Discov 2007; 6(4):287-293.

(4) Mandrekar SJ, Sargent DJ. Predictive biomarker validation in practice: lessons from real trials. Clin Trials 2010.

(5) Sargent DJ, Conley BA, Allegra C, Collette L. Clinical trial designs for predictive marker validation in cancer treatment trials. J Clin Oncol 2005; 23(9):2020-2027.

(6) Simon R. Advances in Clinical Trial Designs for Predictive Biomarker Discovery and Validation. Current Breast Cancer Reports 2009; 1:216-221.

(7) Alymani NA, Smith MD, Williams DJ, Petty RD. Predictive biomarkers for personalised anti-cancer drug use: discovery to clinical implementation. Eur J Cancer 2010; 46(5):869-879.

(8) Jordan VC, Brodie AM. Development and evolution of therapies targeted to the estrogen receptor for the treatment and prevention of breast cancer. Steroids 2007; 72(1):7-25.

(9) Shepard HM, Jin P, Slamon DJ, Pirot Z, Maneval DC. Herceptin. Handb Exp Pharmacol 2008;(181):183-219.

(10) Poste G. Bring on the biomarkers. Nature 2011; 469(7329):156-157.

(11) Holmes MV, Shah T, Vickery C, Smeeth L, Hingorani AD, et al. Fulfilling the promise of personalized medicine? Systematic review and field synopsis of pharmacogenetic studies. PLoS One 2009; 4(12):e7960.

(12) The human genome at ten. Nature 2010; 464(7289):649-650.

(13) Cancer Research UK. Stratified Medicine Programme. http://www.cancerresearchuk.org/science/research/how-we-deliver-our-research/others/by-programme/stratified-medicine-programme/ [ 2011 [cited 2013 Apr. 22];

(14) European public assessment reports. European Medicines Agency [ 2010 [cited 2010 Oct. 19]; Available from: www.ema.europa.eu/ema/index.jsp?curl=/pages/medicines/landing/epar_search.jsp&murl=menus/medicines/medicines.jsp&mid=WC0b01ac058001d124

(15) Pending EC decisions. European Medicines Agency [ 2010 [cited 2010 Oct. 19]; Available from: URL:www.ema.europa.eu/ema/index.jsp?curl=pages/medicines/landing/smop_search.jsp&murl=menus/medicines/medicines.jsp&mid=WC0b01ac058001d127

(16) Simon RM, Paik S, Hayes DF. Use of archived specimens in evaluation of prognostic and predictive biomarkers. J Natl Cancer Inst 2009; 101(21):1446-1452.

For peer review only

(17) European Medicines Agency. Central authorisation of medicines. http://www.ema.europa.eu/ema/index.jsp?curl=pages/about_us/general/general_content_000109.jsp&mid=WC0b01ac0580028a47 [ 2011 [cited 2013 Apr. 22];

(18) European Medicines Agency. Trastuzumab: Summary of product characteristics. http://annonc.oxfordjournals.org/content/12/suppl_1/S57.short [ 2012 [cited 12 A.D. Dec. 20];

(19) European Medicines Agency. How we work. http://www.ema.europa.eu/ema/index.jsp?curl=pages/about_us/general/general_content_000125 jsp&murl=menus/about_us/about_us jsp&mid=WC0b01ac0580028a46 [ 2012 [cited 2012 Feb. 22];

(20) A Giudeline on Summary of Prduct Characteristics. European Medicines Agency [ 2005 [cited 2011 Dec. 15]; Available from: http://ec.europa.eu/health/files/eudralex/vol-2/c/spcguidrev1-oct2005_en.pdf

(21) European Medicines Agency. Orphan Designation. European Medicines Agency [ 2011 [cited 2011 July 28]; Available from: www.ema.europa.eu/ema/index.jsp?curl=pages/regulation/general/general_content_000029.jsp&murl=menus/regulations/regulations.jsp&mid=WC0b01ac05800240ce

(22) Bhathena A, Spear BB. Pharmacogenetics: improving drug and dose selection. Curr Opin Pharmacol 2008; 8(5):639-646.

(23) Bell J. Redefining disease. Harveian Oration 2010.

(24) Barretina J, Caponigro G, Stransky N, Venkatesan K, Margolin AA, et al. The Cancer Cell Line Encyclopedia enables predictive modelling of anticancer drug sensitivity. Nature 2012; 483(7391):603-607.

(25) Garnett MJ, Edelman EJ, Heidorn SJ, Greenman CD, Dastur A, et al. Systematic identification of genomic markers of drug sensitivity in cancer cells. Nature 2012; 483(7391):570-575.

(26) Janes H, Pepe MS, Bossuyt PM, Barlow WE. Measuring the performance of markers for guiding treatment decisions. Ann Intern Med 2011; 154(4):253-259.

For peer review only

## Licence

The Corresponding Author has the right to grant on behalf of all authors and does grant on behalf of all authors, an exclusive licence (or non exclusive for government employees) on a worldwide basis to the BMJ Publishing Group Ltd to permit this article (if accepted) to be published in BMJ editions and any other BMJPGL products and sublicences such use and exploit all subsidiary rights, as set out in our licence.

## Competing interests

All authors declare that they have no financial or non-financial interests that may be relevant to the submitted work.

## Author Contributions

KM, JD, RR and LB designed the review. KM and MB carried out the review. Where needed CC and PJ provided clinical advice. All authors contributed to the interpretation of the results, commented on drafts and accepted the final version of this paper.

The manuscript is an honest, accurate, and transparent account of the study being reported; no important aspects of the study have been omitted; any discrepancies from the study as planned have been explained.

## Data sharing

Data sharing: full dataset available from the corresponding author.

## Ethical Approval

No ethical approval was required, as the study did not involve collection of patient data

## Study funding

This work was funded by the MRC Midlands Hub for Trials Methodology Research at the University of Birmingham (Medical Research Council Grant ID G0800808). The funders had no role in study design, data collection and analysis, decision to publish, or preparation of the manuscript.

For peer review only

**Table 1 Biomarkers predictive of efficacy identified in the review of EMA licensing**

| Biomarker | Indication | Drug |
|---|---|---|
| **ALK gene rearrangement** | Carcinoma, Non-Small-Cell Lung | Crizotinib (Xalkori) |
| **BRAF V600 mutation** | Melanoma | Vemurafenib (Zelboraf) |
| **CCR5 tropism** | HIV Infections | Maraviroc (Celsentri) |
| **CD-33 expression*** | Leukemia, Myeloid, Acute | gemtuzumab ozogamicin (Mylotarg)[‡] |
| **EGFR expression** | Colorectal Neoplasms | Cetuximab (Erbitux) |
| **EGFR expression** | Carcinoma, Non-Small-Cell Lung | Erlotinib (Tarceva) |
| **EGFR mutation** | Carcinoma, Non-Small-Cell Lung | Erlotinib (Tarceva) |
| **EGFR mutation** | Carcinoma, Non-Small-Cell Lung | Gefitinib (Iressa) |
| **EpCAM expression** | CancerAscites | Catumaxomab (Removab) |
| **FIP1L1-PDGFR rearrangement** | Hypereosinophilic Syndrome | Imatinib (Glivec)[#] |
| **G551D mutation in the CFTR gene** | Cystic Fibrosis | Ivacaftor (Kalydeco)[‡] |
| **genotype 1 HCV** | Hepatitis C, Chronic | Boceprevir (Victrelis) |
| **genotype 1 HCV** | Hepatitis C | Telaprevir (Incivo) |
| **HER2 expression** | Breast Neoplasms | Lapatinib (Tyverb) |
| **HER2 expression** | Breast Neoplasms | Trastuzumab (Herceptin) |
| **HER2 expression** | Stomach Neoplasms | Trastuzumab (Herceptin) |
| **HER2 expression** | Breast Neoplasms | Everolimus (Afinitor) |
| **HER2 expression ** ** | Breast Neoplasms | pertuzumab (Perjeta) |
| **Hormone dependency** | Prostatic Neoplasms | Degarelix (Firmagon) |
| **Hormone receptor expression**** | Breast Neoplasms | zoledronic acid (Zometa) |
| **Hormone receptor expression** | Breast Neoplasms | Everolimus (Afinitor) |
| **Kit (CD 117) expression** | Gastrointestinal Stromal Tumors | Imatinib (Glivec)[#] |
| **Kit (D816V) mutation***** | Aggressive Systemic Mastocytosis | Imatinib (Glivec)[#] |
| **KRAS mutation** | Colorectal Neoplasms | Cetuximab (Erbitux) |
| **KRAS mutation** | Colorectal Neoplasms | Panitumumab (Vectibix) |
| **LPL protein detectable** | Hyperlipoproteinemia Type I | alipogene tiparvovec (Glybera)[‡] |
| **oestrogen receptor expression** | Breast Neoplasms | Fulvestrant (Faslodex) |
| **oestrogen receptor expression** | Breast Neoplasms | Toremifene (Fareston) |
| **PDGFR gene rearrangements** | Myelodysplastic-Myeloproliferative Diseases | Imatinib (Glivec)[#] |
| **Philadelphia chromosome** | Precursor Cell Lymphoblastic Leukemia-Lymphoma | Dasatinib (Sprycel)[‡] |
| **Philadelphia chromosome** | Precursor Cell Lymphoblastic Leukemia-Lymphoma | Imatinib (Glivec)[#] |
| **t(15;17) translocation** | Leukemia, Promyelocytic, Acute | arsenic trioxide (Trisenox)[#] |
| **viral resistance mutations***** | HIV Infections | Amprenavir (Agenerase) |
| **viral resistance mutations** | HIV Infections | atazanavir sulphate (Reyataz) |
| **viral resistance mutations** | HIV Infections | Darunavir (Prezista) |
| **viral resistance mutations** | HIV Infections | efavirenz / emtricitabine / tenofovir disoproxil (Atripla) |
| **viral resistance mutations** | HIV Infections | Emtricitabine (Emtriva) |
| **viral resistance mutations** | HIV Infections | emtricitabine / rilpivirine / tenofovir disoproxil (Eviplera) |
| **viral resistance mutations** | HIV Infections | Enfuvirtide (Fuzeon) |

For peer review only

| | | |
|---|---|---|
| **viral resistance mutations** | HIV Infections | fosamprenavir calcium (Telzir) |
| **viral resistance mutations** | HIV Infections | lopinavir / ritonavir (Kaletra) |
| **viral resistance mutations ***** | HIV Infections | Nelfinavir (Viracept) |
| **viral resistance mutations** | HIV Infections | rilpivirine hydrochloride (Edurant) |
| **viral resistance mutations** | HIV Infections | tenofovir disoproxil fumarate (Viread) |
| **viral resistance mutations** | HIV Infections | Tipranavir (Aptivus) |

* refused **pending ***withdrawn
‡ drug designated an orphan medicine, # orphan designation has been removed at the end of exclusivity period

**Table 2 Biomarkers predictive of toxicity identified in the review of EMA licensing**

| Biomarker | Indication | Drug |
|---|---|---|
| **DPD deficiency** | Colorectal Neoplasms<br>Colonic Neoplasms<br>Stomach Neoplasms<br>Breast Neoplasms | Capecitabine (Xeloda and generic drugs: Capecitabine Accord; Capecitabine Krka; Capecitabine Medac; Capecitabine Teva) |
| **DPD deficiency** | Stomach Neoplasms | tegafur / gimeracil / oteracil (Teysuno) |
| **HLA-B*5701 allele** | HIV Infections | Abacavir (Kivexa; Trizivir; Ziagen)* |
| **NADPH reductase deficiency** | Methemoglobinemia | Methylthioninium chloride (Methylthioninium chloride Proveblue) |

* HLA-B*5701 allele is predictive of hypersensitivity to abacavir, which is present in three three drugs: Kivexa (abacavir / lamivudine); Trizivir (abacavir / lamivudine / zidovudine); Ziagen (abacavir)

For peer review only

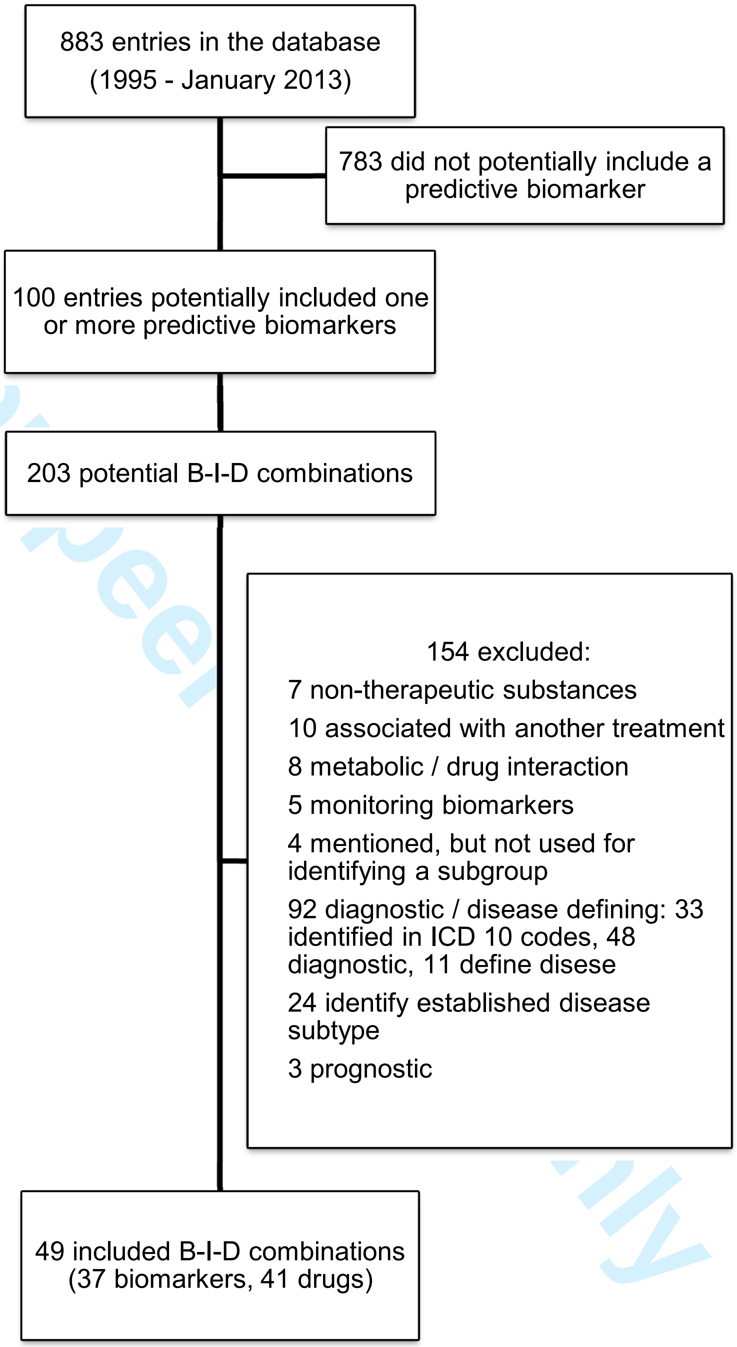


**Figure 1 Flow diagram for the systematic review of predictive biomarkeres in EMA licensig**

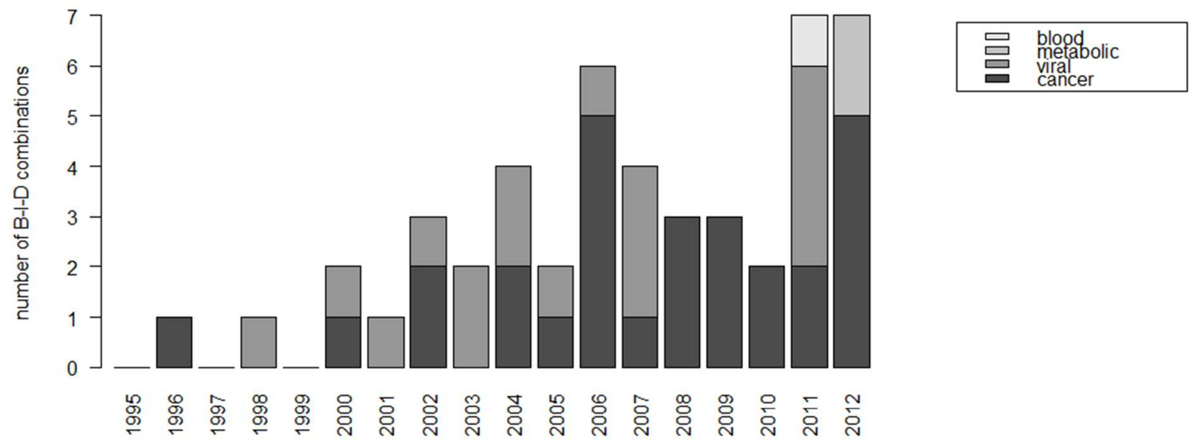


**Figure 2 Number of new B-I-D combinations considered each year by disease area**

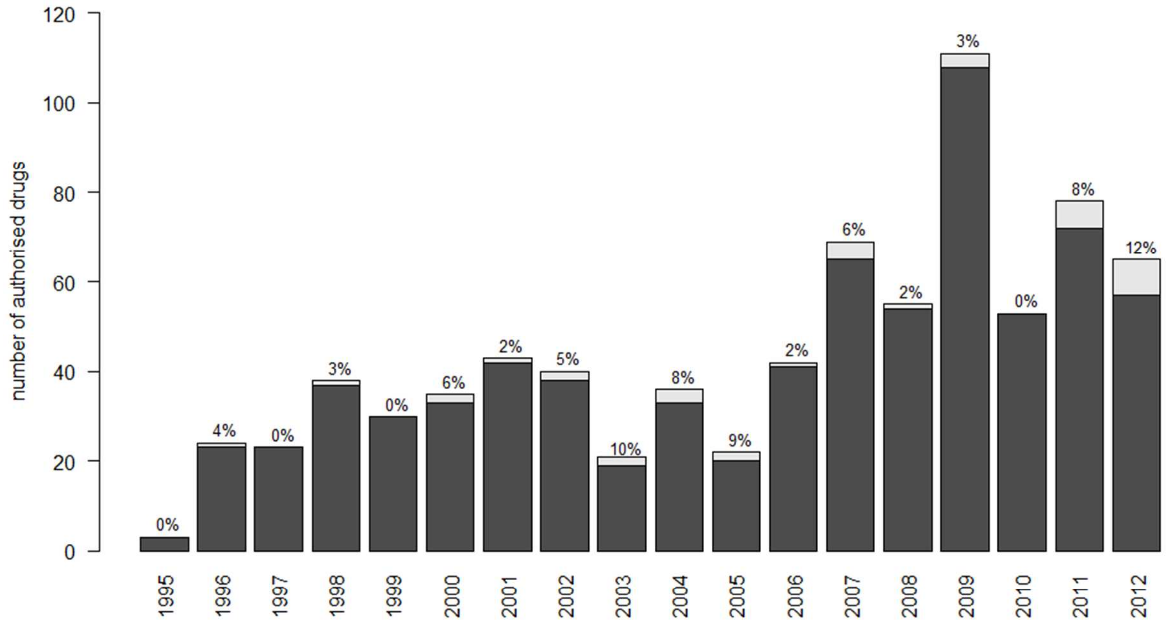


Data for 2012 include 4 generic drugs (capecitabine)

**Figure 3 New drugs authorised each year with and without a predictive biomarker in the indication or contraindication (excludes biomarkers added after the drug was initially licensed)**

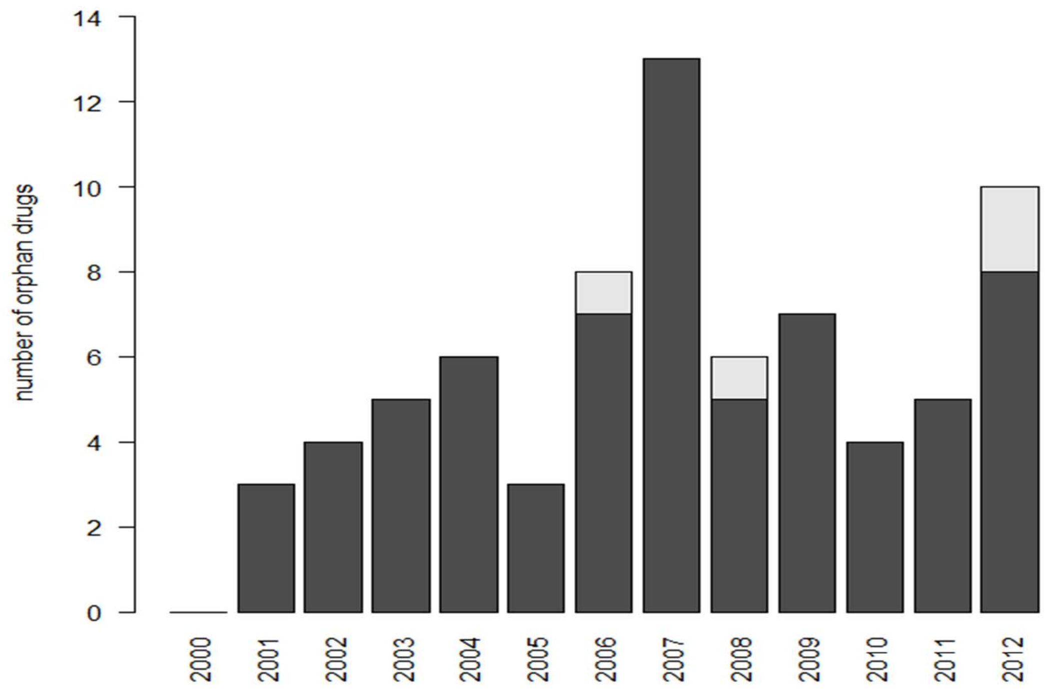


**Figure 4 New drugs orphan authorised each year with and without a predictive biomarker in the indication or contraindication (excludes biomarkers added after the drug was initially licensed)**

# PRISMA 2009 Checklist

| Section/topic | # | Checklist item | Reported on page # |
|---|---|---|---|
| **TITLE** | | | |
| Title | 1 | Identify the report as a systematic review, meta-analysis, or both. | 1 |
| **ABSTRACT** | | | |
| Structured summary | 2 | Provide a structured summary including, as applicable: background; objectives; data sources; study eligibility criteria, participants, and interventions; study appraisal and synthesis methods; results; limitations; conclusions and implications of key findings; systematic review registration number. | 2 |
| **INTRODUCTION** | | | |
| Rationale | 3 | Describe the rationale for the review in the context of what is already known. | 3 |
| Objectives | 4 | Provide an explicit statement of questions being addressed with reference to participants, interventions, comparisons, outcomes, and study design (PICOS). | 3 |
| **METHODS** | | | |
| Protocol and registration | 5 | Indicate if a review protocol exists, if and where it can be accessed (e.g., Web address), and, if available, provide registration information including registration number. | 2 |
| Eligibility criteria | 6 | Specify study characteristics (e.g., PICOS, length of follow-up) and report characteristics (e.g., years considered, language, publication status) used as criteria for eligibility, giving rationale. | 3-4 |
| Information sources | 7 | Describe all information sources (e.g., databases with dates of coverage, contact with study authors to identify additional studies) in the search and date last searched. | 3-4 |
| Search | 8 | Present full electronic search strategy for at least one database, including any limits used, such that it could be repeated. | 3 |
| Study selection | 9 | State the process for selecting studies (i.e., screening, eligibility, included in systematic review, and, if applicable, included in the meta-analysis). | 4 |
| Data collection process | 10 | Describe method of data extraction from reports (e.g., piloted forms, independently, in duplicate) and any processes for obtaining and confirming data from investigators. | 4-5 |
| Data items | 11 | List and define all variables for which data were sought (e.g., PICOS, funding sources) and any assumptions and simplifications made. | 4-5 |
| Risk of bias in individual studies | 12 | Describe methods used for assessing risk of bias of individual studies (including specification of whether this was done at the study or outcome level), and how this information is to be used in any data synthesis. | Not applicable |
| Summary measures | 13 | State the principal summary measures (e.g., risk ratio, difference in means). | Not applicable |

For peer review only

# PRISMA 2009 Checklist

| | | | |
|---|---|---|---|
| Synthesis of results | 14 | Describe the methods of handling data and combining results of studies, if done, including measures of consistency (e.g., $I^2$) for each meta-analysis. | Not applicable |



| Section/topic | # | Checklist item | Reported on page # |
|---|---|---|---|
| Risk of bias across studies | 15 | Specify any assessment of risk of bias that may affect the cumulative evidence (e.g., publication bias, selective reporting within studies). | Not applicable |
| Additional analyses | 16 | Describe methods of additional analyses (e.g., sensitivity or subgroup analyses, meta-regression), if done, indicating which were pre-specified. | Not applicable |
| **RESULTS** | | | |
| Study selection | 17 | Give numbers of studies screened, assessed for eligibility, and included in the review, with reasons for exclusions at each stage, ideally with a flow diagram. | 13 |
| Study characteristics | 18 | For each study, present characteristics for which data were extracted (e.g., study size, PICOS, follow-up period) and provide the citations. | Not applicable |
| Risk of bias within studies | 19 | Present data on risk of bias of each study and, if available, any outcome level assessment (see item 12). | Not applicable |
| Results of individual studies | 20 | For all outcomes considered (benefits or harms), present, for each study: (a) simple summary data for each intervention group (b) effect estimates and confidence intervals, ideally with a forest plot. | Not applicable |
| Synthesis of results | 21 | Present results of each meta-analysis done, including confidence intervals and measures of consistency. | Not applicable |
| Risk of bias across studies | 22 | Present results of any assessment of risk of bias across studies (see Item 15). | Not applicable |
| Additional analysis | 23 | Give results of additional analyses, if done (e.g., sensitivity or subgroup analyses, meta-regression [see Item 16]). | Not applicable |
| **DISCUSSION** | | | |
| Summary of evidence | 24 | Summarize the main findings including the strength of evidence for each main outcome; consider their relevance to key groups (e.g., healthcare providers, users, and policy makers). | 6 |
| Limitations | 25 | Discuss limitations at study and outcome level (e.g., risk of bias), and at review-level (e.g., incomplete retrieval of identified research, reporting bias). | 6 |
| Conclusions | 26 | Provide a general interpretation of the results in the context of other evidence, and implications for future research. | 6-7 |
| **FUNDING** | | | |

For peer review only

# PRISMA 2009 Checklist

| | | | |
|---|---|---|---|
| Funding | 27 | Describe sources of funding for the systematic review and other support (e.g., supply of data); role of funders for the systematic review. | 10 |

*From:* Moher D, Liberati A, Tetzlaff J, Altman DG, The PRISMA Group (2009). Preferred Reporting Items for Systematic Reviews and Meta-Analyses: The PRISMA Statement. PLoS Med 6(6): e1000097. doi:10.1371/journal.pmed1000097

For more information, visit: **www.prisma-statement.org**.



For peer review only

For peer review only - http://bmjopen.bmj.com/site/about/guidelines.xhtml

# Stratified medicine in European Medicines Agency licensing: a systematic review of predictive biomarkers


| | |
|---|---|
| Journal: | *BMJ Open* |
| Manuscript ID: | bmjopen-2013-004188.R1 |
| Article Type: | Research |
| Date Submitted by the Author: | 06-Dec-2013 |


| | |
|---|---|
| Complete List of Authors: | Malottki, Kinga; University of Birmingham , Public Health, Epidemiology and Biostatistics<br>Biswas, Mousumi; University of Bristol, School of Social and Community Medicine<br>Deeks, Jon; University of Birmingham, Public Health Epidemiology and Biostatistics<br>Riley, Richard; University of Birmingham, Public Health Epidemiology and Biostatistic<br>Craddock, Charles; Queen Elizabeth Hospital, Centre for Clinical Haematology<br>Johnson, Philip; University of Liverpool, Clatterbridge Cancer Centre NHS Foundation Trust<br>Billingham, Lucinda; University of Birmingham, Public Health Epidemiology and Biostatistics |


| | |
|---|---|
| <b>Primary Subject Heading</b>: | Pharmacology and therapeutics |
| Secondary Subject Heading: | Public health |
| Keywords: | THERAPEUTICS, Molecular aspects < ONCOLOGY, Molecular diagnostics < INFECTIOUS DISEASES |


SCHOLARONE™
Manuscripts

# Stratified medicine in European Medicines Agency licensing: a systematic review of predictive biomarkers

Kinga Malottki*, Mousumi Biswas, Jonathan J. Deeks, Richard D. Riley, Charles Craddock, Philip Johnson, Lucinda Billingham
* corresponding author

Kinga Malottki
Research Fellow
MRC Midland Hub for Trials Methodology Research, University of Birmingham, Birmingham, B15 2TT, UK
k.malottki@bham.ac.uk

Mousumi Biswas
Research Associate
The Discovery Research Programme, School of Social and Community Medicine, University of Bristol, Bristol, BS8 2PS, UK

Jonathan J. Deeks
Professor
Public Health, Epidemiology and Biostatistics, School of Health and Population Sciences, University of Birmingham, Birmingham, B15 2TT, UK
MRC Midland Hub for Trials Methodology Research, University of Birmingham, Birmingham, B15 2TT, UK

Richard D. Riley
Reader
Public Health, Epidemiology and Biostatistics, School of Health and Population Sciences, University of Birmingham, Birmingham, B15 2TT, UK
MRC Midland Hub for Trials Methodology Research, University of Birmingham, Birmingham, B15 2TT, UK

Charles Craddock
Professor
Centre for Clinical Haematology, Queen Elizabeth Hospital, Birmingham, B15 2TH, UK

Philip Johnson
Professor
University of Liverpool & Clatterbridge Cancer Centre NHS Foundation Trust, Liverpool L69 3GA, UK

Lucinda Billingham
Professor
MRC Midland Hub for Trials Methodology Research, University of Birmingham, Birmingham, B15 2TT, UK
Cancer Research UK Clinical Trials Unit, University of Birmingham, Birmingham, B15 2TT, UK

For peer review only

**Abstract**

**Objectives** Stratified medicine is often heralded as the future of clinical practice. Key part of stratified medicine is the use of predictive biomarkers, which identify patient subgroups most likely to benefit (or least likely to experience harm) from an intervention. We investigated how many and what predictive biomarkers are currently included in European Medicines Agency licensing.

**Methods and eligibility criteria** Indications and contraindications of all drugs considered by the EMA and published on their website were screened to identify predictive biomarkers. For all included Biomarker-Indication-Drug (B-I-D) combinations data was collected on: the type of the biomarker, whether it selected a subgroup of patients based on efficacy or toxicity, therapeutic area, marketing status, date of licensing decision, date of inclusion of the biomarker in the indication or contraindication, and on orphan designation.

**Results** 49 B-I-D combinations were identified over 16 years, which included 37 biomarkers and 41 different drugs. All identified biomarkers were molecular. Six drugs (relating to 10 B-I-D combinations) had an orphan designation at the time of licensing. The identified B-I-D combinations were mainly used in cancer and HIV treatment, but also in hepatitis C and three other indications (cystic fibrosis, hyperlipoproteinemia type I, and methemoglobinemia). In 45 B-I-D combinations biomarkers were used as predictive of drug efficacy and in four of drug toxicity. It appeared that there was an increase in the number of B-I-D combinations introduced each year, however the numbers were too small to identify any trends.

**Conclusions** Given the large body of literature documenting research into potential predictive biomarkers and extensive investment into stratified medicine, we identified relatively few predictive biomarkers included in licensing. These were also limited to a small number of clinical areas. This might suggest a need for improvement in methods of translation from laboratory findings to clinical practice.

For peer review only

## Article summary

### Article focus

- To identify predictive biomarkers included in European Medicines Agency licensing
- For identified biomarkers, to investigate their type, the clinical areas in which these biomarkers are used and possible trends over time with regard to the number of new predictive biomarkers considered each year

### Key messages

- 49 Biomarker-Indication-Drug (B-I-D) combinations were identified over 16 years, which included 37 biomarkers and 41 different drugs. There appeared to be an increase in the number of B-I-D combinations introduced each year, however the numbers were too small to identify any trends.
- All identified biomarkers were molecular. They were mainly used in cancer and HIV treatment, but also in hepatitis C and three other indications (cystic fibrosis, hyperlipoproteinemia type I, and methemoglobinemia).

### Strengths and limitations of this study

- Our research, to our knowledge, provides the first indication of the number and nature of predictive biomarkers included in licensing in Europe using systematic review methodology.
- It is likely that the 49 identified B-I-D combinations do not represent a complete list of predictive biomarkers used in practice, as some could have been considered by national regulatory agencies, particularly for drugs considered before EMA was established in 1995.

For peer review only

## Introduction

Drugs are rarely effective in all patients and may be associated with serious adverse events.[1] The challenge of stratified medicine is to identify predictive biomarkers that identify patient subgroups (or strata) with a differential therapeutic response to a linked intervention, allowing more appropriate and effective use of interventions to maximise patient benefit and minimise the occurrence of serious adverse events.[2;3] Predictive biomarkers are defined particular to a treatment for a condition, where biomarker values are associated with differential efficacy or toxicity of that treatment.[4-7] The use of predictive biomarkers promises a more appropriate choice of treatment: it can also help to rationalise funding decisions, avoiding costs of futile treatment and of adverse events. However the additional cost of measuring the marker has to be taken into account. Examples of predictive biomarkers include tamoxifen use in breast cancer, which is prescribed to women who are oestrogen receptor positive,[8] and trastuzumab which is prescribed to those with HER2 overexpression in their tumour.[9]

There is a large body of literature documenting research into potential predictive biomarkers,[10;11] and millions of pounds have been invested into stratified medicine, both in industry and through programs from funding bodies such as the Medical Research Council[12] and Cancer Research UK.[13] We aimed to investigate if this interest in developing stratified medicines has led to production of biomarker-treatment combinations ready for use in clinical practice. To explore this question, we have undertaken a systematic review of predictive biomarkers reported in licensing decisions of the European Medicines Agency (EMA).

In our review we aimed to find out how many of the indications and contraindications considered by the EMA define a patient population using a predictive biomarker. We were also interested in the disease areas where predictive biomarkers have been used and any trend over time. It has been hypothesised that stratified medicine has not been implemented in practice as much as expected. This paper provides evidence of the impact of stratified medicine research to date and if less than expected, then this will highlight the need to review the underlying reasons and address the problems.

## Methods

We defined a Biomarker-Indication-Drug (B-I-D) combination as the unit of our analysis, relating to the use of a predictive biomarker with a particular drug for a particular condition or disease.[4-7] For toxicity biomarkers where the a biomarkers of drug toxicity may be used in more than one disease area we grouped these into one B-I-D combination.

All drugs listed on the EMA website in either European Public Assessment Reports or Pending Decisions[14;15] (accessed on the 17th of January 2013) were evaluated, together with their indications and contraindications.

Our inclusion criteria were that the biomarker had to:

For peer review only

(i) be used in the indication and/or contraindication of the drug,
(ii) be associated with a particular treatment,
(iii) identify a subgroup of patients with a particular disease eligible for treatment with the drug.

We excluded biomarkers:

(i) associated with a non-therapeutic substance (for example vaccines),
(ii) not used as predictive, including:
   - used for diagnosis, screening or forming part of the disease definition (already established for defining a disease) or established disease subtype,
   - prognostic only (associated with outcome regardless of treatment and not predictive of treatment response[16])
(iii) associated with another treatment (for example the biomarker was not associated with the differential efficacy or toxicity of the drug of interest, but another drug given in combination with the drug of interest).

We have reviewed EMA licensing, as in Europe a centralised drug evaluation by the EMA is required for drugs for treatment of a number of conditions, drugs obtained from biotechnology processes and all drugs used for rare conditions (orphan medicines). Companies can also apply for a centralised marketing authorisation of other drugs.[17] Although the EMA does not license biomarkers, it evaluates drugs in groups defined by predictive biomarkers (for example trastuzumab is licensed for use in HER2 overexpressing breast cancer patients).[18] Our approach is likely to give a broad overview of the impact of predictive biomarkers on treatment selection since 1995 (when EMA was established[19]).

We created a database of all drugs in the EMA database including the drug name, licensing status, indication and contraindication. In the first stage of screening all database entries were screened by two independent reviewers (MB and KM) to identify those potentially including a predictive biomarker in the indication or contraindication. If an entry was identified by at least one of the reviewers as potentially relevant, it was included in the second stage of screening.

In the second stage of screening, a list of potential B-I-D combinations was created based on the entries identified in the first stage. The list of potential B-I-D combinations was assessed by two independent reviewers (MB and KM) using full inclusion/exclusion criteria, based on the information in the Summary of Product Characteristics (which sets out the position of the drug obtained in the assessment process and summarises its properties and clinical use together with the clinical trial evidence that was considered by the EMA)[20] , the Scientific Discussion (which discusses the properties and clinical evidence in more detail) and additional information from targeted internet searches and expert advice if necessary. Any disagreements were resolved by discussion.

For the included B-I-D combinations data was collected on: the type of the biomarker used as predictive, whether it selected a subgroup of patients based on efficacy or toxicity, therapeutic area, marketing

For peer review only

status, date of licensing decision, date of inclusion of the biomarker in the indication or contraindication, and on orphan designation (granted to drugs intended for the treatment of a life-threatening or chronically debilitating condition which is either affecting no more than 5 in 10,000 people in the EU or when the revenue is unlikely to cover the investment in drug development[21]). To provide a context for our review, we have also collected data on the total number of drugs licensed each year with and without an orphan designation.

**Results**

Across the 18 year period (1995-2012) we identified 49 B-I-D combinations, including 37 biomarkers and 41 different drugs. The details of the review process are presented in Figure 1~~Figure 1~~. Most of the drugs were authorised, the exceptions being:

- Gemtuzumab ozogamicin (refused)
- Zeldoronic acid (pending)
- Imatinib in the indication for aggressive systemic mastocytosis (withdrawn)
- Amprnavir (withdrawn)
- Nelfinavir (withdrawn)

The number of new B-I-D combinations considered by the EMA each year has increased overall from zero or one per year in the late nineties, to a maximum of 7 in each of 2011 and 2012 as shown in Figure 2~~Figure 2~~. This was however not a steady increase, as the number of B-I-D combinations considered by the EMA showed fluctuation between 2000 and 2006, a decrease between 2006 and 2010, followed by an increase in the number in 2011 and 2012. A predictive biomarker was included in the indication or contraindication at the time when the drug was first licensed for 35 drugs (for one (capecitabine) the date of inclusion of the biomarker was unclear from the documentation, for the remaining drugs the time from the initial licensing decision to the inclusion of a predictive biomarker ranged from one to ten years). The proportion of first licensing decision of all new drugs that included a predictive biomarker increased over time and was close to 10% in 2003, 2004, 2005, 2011 and 2012 (Figure 3).

Six drugs associated with a predictive biomarker had an orphan designation at the time of licensing, however for two it was removed at the end of exclusivity period (details reported in

Table 1~~Table 1~~). One of the six drugs (imatinib) was associated with five different predictive biomarkers in five different indications (Figure 4).

The identified predictive biomarkers were all molecular. Thirty-three biomarkers were used to predict treatment efficacy (details reported in

Table 1~~Table 1~~) and only four to predict toxicity (Table 2~~Table 2~~).

For peer review only

Most of the biomarkers were included in indications and contraindications of cancer treatments (26 B-I-D combinations) and viral diseases, mainly HIV (17 B-I-D combinations). The remaining biomarkers were used to stratify metabolic and blood disorders (cystic fibrosis, hyperlipoproteinemia type I, and methemoglobinemia) and appeared in the last two years (Figure 2Figure 2).

**Discussion**

Stratified medicine is promoted as key to the future of medicine, and is currently one of the most active areas of clinical research. To our knowledge this review provides the first indication of the number and nature of predictive biomarkers included in licensing in Europe based on the drug indications and contraindications on the EMA website. Forty nine B-I-D combinations were identified. All identified biomarkers were molecular. The identified B-I-D combinations were mainly used in cancer and HIV treatment, with only five used in other disease areas.

It is likely that the 49 identified B-I-D combinations from the EMA database do not represent a complete list of the predictive biomarkers used in practice as some predictive biomarkers could have been considered by national regulatory agencies, particularly for drugs considered before EMA was established in 1995. Also EMA licensing is not compulsory for some disease areas, such as mental health. However a number of drugs with indications in depression of schizophrenia have been considered by the EMA. Therefore we believe that although our approach might not provide a complete list of all predictive biomarkers used in Europe, relatively few are likely to have been omitted, particularly from recent years.[19] The fact that some of the identified B-I-D combinations included biomarkers introduced to an indication of an already licensed drug suggests that at least to some extent we have captured stratification occurring after the initial licensing of a drug. However the actual extent to which this takes place in clinical practice is difficult to evaluate.

Several types of biomarkers were excluded. We did not include biomarkers used for dose adjustments as they do not directly predict efficacy or toxicity (although inappropriate dose adjustment could limit the treatment efficacy or cause adverse events).[22] We also only investigated biomarkers associated with drug treatments. Other biomarkers may be used in practice with non-drug treatments (for example radiotherapy).

The definition of a predictive biomarker can be difficult to apply, as over time predictive biomarkers may become part of a redefinition of the disease or subtype of disease[23] and be classed as diagnostic tests. In our evaluation we excluded diagnostic biomarkers (for example these included factor IX deficiency, or genetic testing for familial lipoprotein lipase deficiency), and biomarkers used to identify an established subtype of a disease (mainly ST segment elevation and non-ST segment elevation myocardial infarction).

For peer review only

The spectrum of diseases where predictive biomarkers have been successfully developed is relatively narrow. This suggests a possible need for more research in other clinical areas. Also the vast majority of the B-I-D combinations were associated with treatment efficacy and only four with toxicity. As adverse events associated with some treatments could be potentially serious and the possibility to screen out patients at high risk prior to commencing treatment would be beneficial. A proportion of the drugs with an associated predictive biomarker identified in our review had an orphan designation. This seems surprising, as convincing evidence to support the use of a drug in a subgroup of patients with a rare condition might be difficult to obtain, due to the small numbers of patients available to test the hypotheses.

It is difficult to provide accurate estimates of the extent of research into potential predictive biomarkers, however it has been suggested in 2011 that the number of publications on different biomarkers (not only predictive) was in the area of 15 000.[10] Another paper published in 2009, which reviewed genetic markers evaluated as potential predictors of response to treatment, found that 541 different genes were investigated as potential predictive biomarkers in 1 668 papers.[11] It can be reasonably expected that this number largely increased since these papers were published. Our review shows that few predictive biomarkers have been included in licensing relative to this large body of literature documenting numerous potential predictive biomarkers. Therefore, in spite of the substantial investment in research, the promise of stratified medicine is not yet being realised to a large extent. The reasons for this might include poor translation of findings of laboratory studies into clinical context, or the failure to identify effective predictive biomarkers and treatments. Even though it is becoming easier and cheaper to gather huge sets of genomic data, its interpretation is challenging, which can potentially hinder translational research. Recognising this, initiatives have been undertaken both in the USA (National Institutes of Health and the FDA) and UK (Medical Research Council) to promote the translation of basic research into clinical practice.[12] Also the availability of datasets such as the Cancer Cell Line Encyclopaedia and a similar UK initiative might contribute to the faster progress of stratified medicine.[24;25] The relatively small number of predictive biomarkers identified in licensing might also indicate the need for more sound methodological standards for biomarker discovery and development.[26]

For peer review only

**Licence**

The Corresponding Author has the right to grant on behalf of all authors and does grant on behalf of all authors, an exclusive licence (or non exclusive for government employees) on a worldwide basis to the BMJ Publishing Group Ltd to permit this article (if accepted) to be published in BMJ editions and any other BMJPGL products and sublicences such use and exploit all subsidiary rights, as set out in our licence.

**Competing interests**

All authors declare that they have no financial or non-financial interests that may be relevant to the submitted work.

**Author Contributions**

KM, JD, RR and LB designed the review. KM and MB carried out the review. Where needed CC and PJ provided clinical advice. All authors contributed to the interpretation of the results, commented on drafts and accepted the final version of this paper.

The manuscript is an honest, accurate, and transparent account of the study being reported; no important aspects of the study have been omitted; any discrepancies from the study as planned have been explained.

**Data sharing**

Data sharing: full dataset available from the corresponding author.

**Ethical Approval**

No ethical approval was required, as the study did not involve collection of patient data

**Study funding**

This work was funded by the MRC Midlands Hub for Trials Methodology Research at the University of Birmingham (Medical Research Council Grant ID G0800808). The funders had no role in study design, data collection and analysis, decision to publish, or preparation of the manuscript.

For peer review only

## References


(1) Xinghua Hu S, Foster T, Kieffaber A. Pharmacogenomics and personalized medicine: mapping future value creation. BioTechniques 2005; 39(4).

(2) Aroon DH, Danielle AvdW, Richard DR, et al. Prognosis research strategy (PROGRESS) 4: Stratified medicine research. BMJ 2013; 346.

(3) Trusheim MR, Berndt ER, Douglas FL. Stratified medicine: strategic and economic implications of combining drugs and clinical biomarkers. Nat Rev Drug Discov 2007; 6(4):287-293.

(4) Mandrekar SJ, Sargent DJ. Predictive biomarker validation in practice: lessons from real trials. Clin Trials 2010.

(5) Sargent DJ, Conley BA, Allegra C, et al. Clinical trial designs for predictive marker validation in cancer treatment trials. J Clin Oncol 2005; 23(9):2020-2027.

(6) Simon R. Advances in Clinical Trial Designs for Predictive Biomarker Discovery and Validation. Current Breast Cancer Reports 2009; 1:216-221.

(7) Alymani NA, Smith MD, Williams DJ, et al. Predictive biomarkers for personalised anti-cancer drug use: discovery to clinical implementation. Eur J Cancer 2010; 46(5):869-879.

(8) Jordan VC, Brodie AM. Development and evolution of therapies targeted to the estrogen receptor for the treatment and prevention of breast cancer. Steroids 2007; 72(1):7-25.

(9) Shepard HM, Jin P, Slamon DJ, et al. Herceptin. Handb Exp Pharmacol 2008;(181):183-219.

(10) Poste G. Bring on the biomarkers. Nature 2011; 469(7329):156-157.

(11) Holmes MV, Shah T, Vickery C, et al. Fulfilling the promise of personalized medicine? Systematic review and field synopsis of pharmacogenetic studies. PLoS One 2009; 4(12):e7960.

(12) The human genome at ten. Nature 2010; 464(7289):649-650.

(13) Cancer Research UK. Stratified Medicine Programme. http://www.cancerresearchuk.org/science/research/how-we-deliver-our-research/others/by-programme/stratified-medicine-programme/ [ 2011 [cited 2013 Apr. 22];

(14) European public assessment reports. European Medicines Agency [ 2010 [cited 2010 Oct. 19]; Available from: www.ema.europa.eu/ema/index.jsp?curl=/pages/medicines/landing/epar_search.jsp&murl=menus/medicines/medicines.jsp&mid=WC0b01ac058001d124

(15) Pending EC decisions. European Medicines Agency [ 2010 [cited 2010 Oct. 19]; Available from: URL:www.ema.europa.eu/ema/index.jsp?curl=pages/medicines/landing/smop_search.jsp&murl=menus/medicines/medicines.jsp&mid=WC0b01ac058001d127

(16) Simon RM, Paik S, Hayes DF. Use of archived specimens in evaluation of prognostic and predictive biomarkers. J Natl Cancer Inst 2009; 101(21):1446-1452.


For peer review only

(17) European Medicines Agency. Central authorisation of medicines. http://www.ema.europa.eu/ema/index.jsp?curl=pages/about_us/general/general_content_000109.jsp&mid=WC0b01ac0580028a47 [ 2011 [cited 2013 Apr. 22];

(18) European Medicines Agency. Trastuzumab: Summary of product characteristics. http://annonc.oxfordjournals.org/content/12/suppl_1/S57.short [ 2012 [cited 12 A.D. Dec. 20];

(19) European Medicines Agency. How we work. http://www.ema.europa.eu/ema/index.jsp?curl=pages/about_us/general/general_content_000125jsp&murl=menus/about_us/about_us jsp&mid=WC0b01ac0580028a46 [ 2012 [cited 2012 Feb. 22];

(20) A Giudeline on Summary of Prduct Characteristics. European Medicines Agency [ 2005 [cited 2011 Dec. 15]; Available from: http://ec.europa.eu/health/files/eudralex/vol-2/c/spcguidrev1-oct2005_en.pdf

(21) European Medicines Agency. Orphan Designation. European Medicines Agency [ 2011 [cited 2011 July 28]; Available from: www.ema.europa.eu/ema/index.jsp?curl=pages/regulation/general/general_content_000029.jsp&murl=menus/regulations/regulations.jsp&mid=WC0b01ac05800240ce

(22) Bhathena A, Spear BB. Pharmacogenetics: improving drug and dose selection. Curr Opin Pharmacol 2008; 8(5):639-646.

(23) Bell J. Redefining disease. Harveian Oration 2010.

(24) Barretina J, Caponigro G, Stransky N, et al. The Cancer Cell Line Encyclopedia enables predictive modelling of anticancer drug sensitivity. Nature 2012; 483(7391):603-607.

(25) Garnett MJ, Edelman EJ, Heidorn SJ, Greenman CD, Dastur A, et al. Systematic identification of genomic markers of drug sensitivity in cancer cells. Nature 2012; 483(7391):570-575.

(26) Janes H, Pepe MS, Bossuyt PM, et al. Measuring the performance of markers for guiding treatment decisions. Ann Intern Med 2011; 154(4):253-259.

For peer review only

**Table 1 Biomarkers predictive of efficacy identified in the review of EMA licensing**

| Biomarker | Indication | Drug |
|---|---|---|
| **ALK gene rearrangement** | Carcinoma, Non-Small-Cell Lung | Crizotinib (Xalkori) |
| **BRAF V600 mutation** | Melanoma | Vemurafenib (Zelboraf) |
| **CCR5 tropism** | HIV Infections | Maraviroc (Celsentri) |
| **CD-33 expression*** | Leukemia, Myeloid, Acute | gemtuzumab ozogamicin (Mylotarg)[‡] |
| **EGFR expression** | Colorectal Neoplasms | Cetuximab (Erbitux) |
| **EGFR expression** | Carcinoma, Non-Small-Cell Lung | Erlotinib (Tarceva) |
| **EGFR mutation** | Carcinoma, Non-Small-Cell Lung | Erlotinib (Tarceva) |
| **EGFR mutation** | Carcinoma, Non-Small-Cell Lung | Gefitinib (Iressa) |
| **EpCAM expression** | CancerAscites | Catumaxomab (Removab) |
| **FIP1L1-PDGFR rearrangement** | Hypereosinophilic Syndrome | Imatinib (Glivec)[#] |
| **G551D mutation in the CFTR gene** | Cystic Fibrosis | Ivacaftor (Kalydeco)[‡] |
| **genotype 1 HCV** | Hepatitis C, Chronic | Boceprevir (Victrelis) |
| **genotype 1 HCV** | Hepatitis C | Telaprevir (Incivo) |
| **HER2 expression** | Breast Neoplasms | Lapatinib (Tyverb) |
| **HER2 expression** | Breast Neoplasms | Trastuzumab (Herceptin) |
| **HER2 expression** | Stomach Neoplasms | Trastuzumab (Herceptin) |
| **HER2 expression** | Breast Neoplasms | Everolimus (Afinitor) |
| **HER2 expression **** | Breast Neoplasms | pertuzumab (Perjeta) |
| **Hormone dependency** | Prostatic Neoplasms | Degarelix (Firmagon) |
| **Hormone receptor expression**** | Breast Neoplasms | zoledronic acid (Zometa) |
| **Hormone receptor expression** | Breast Neoplasms | Everolimus (Afinitor) |
| **Kit (CD 117) expression** | Gastrointestinal Stromal Tumors | Imatinib (Glivec)[#] |
| **Kit (D816V) mutation***** | Aggressive Systemic Mastocytosis | Imatinib (Glivec)[#] |
| **KRAS mutation** | Colorectal Neoplasms | Cetuximab (Erbitux) |
| **KRAS mutation** | Colorectal Neoplasms | Panitumumab (Vectibix) |
| **LPL protein detectable** | Hyperlipoproteinemia Type I | alipogene tiparvovec (Glybera)[‡] |
| **oestrogen receptor expression** | Breast Neoplasms | Fulvestrant (Faslodex) |
| **oestrogen receptor expression** | Breast Neoplasms | Toremifene (Fareston) |
| **PDGFR gene rearrangements** | Myelodysplastic-Myeloproliferative Diseases | Imatinib (Glivec)[#] |
| **Philadelphia chromosome** | Precursor Cell Lymphoblastic Leukemia-Lymphoma | Dasatinib (Sprycel)[‡] |
| **Philadelphia chromosome** | Precursor Cell Lymphoblastic Leukemia-Lymphoma | Imatinib (Glivec)[#] |
| **t(15;17) translocation** | Leukemia, Promyelocytic, Acute | arsenic trioxide (Trisenox)[#] |
| **viral resistance mutations***** | HIV Infections | Amprenavir (Agenerase) |
| **viral resistance mutations** | HIV Infections | atazanavir sulphate (Reyataz) |
| **viral resistance mutations** | HIV Infections | Darunavir (Prezista) |
| **viral resistance mutations** | HIV Infections | efavirenz / emtricitabine / tenofovir disoproxil (Atripla) |
| **viral resistance mutations** | HIV Infections | Emtricitabine (Emtriva) |
| **viral resistance mutations** | HIV Infections | emtricitabine / rilpivirine / tenofovir |

For peer review only

| | | |
|---|---|---|
| | | disoproxil (Eviplera) |
| **viral resistance mutations** | HIV Infections | Enfuvirtide (Fuzeon) |
| **viral resistance mutations** | HIV Infections | fosamprenavir calcium (Telzir) |
| **viral resistance mutations** | HIV Infections | lopinavir / ritonavir (Kaletra) |
| **viral resistance mutations ***** | HIV Infections | Nelfinavir (Viracept) |
| **viral resistance mutations** | HIV Infections | rilpivirine hydrochloride (Edurant) |
| **viral resistance mutations** | HIV Infections | tenofovir disoproxil fumarate (Viread) |
| **viral resistance mutations** | HIV Infections | Tipranavir (Aptivus) |

* refused **pending ***withdrawn
‡ drug designated an orphan medicine, # orphan designation has been removed at the end of exclusivity period

**Table 2 Biomarkers predictive of toxicity identified in the review of EMA licensing**

| Biomarker | Indication | Drug |
|---|---|---|
| **DPD deficiency** | Colorectal Neoplasms<br>Colonic Neoplasms<br>Stomach Neoplasms<br>Breast Neoplasms | Capecitabine (Xeloda and generic drugs: Capecitabine Accord; Capecitabine Krka; Capecitabine Medac; Capecitabine Teva) |
| **DPD deficiency** | Stomach Neoplasms | tegafur / gimeracil / oteracil (Teysuno) |
| **HLA-B*5701 allele** | HIV Infections | Abacavir (Kivexa; Trizivir; Ziagen)* |
| **NADPH reductase deficiency** | Methemoglobinemia | Methylthioninium chloride (Methylthioninium chloride Proveblue) |

* HLA-B*5701 allele is predictive of hypersensitivity to abacavir, which is present in three three drugs: Kivexa (abacavir / lamivudine); Trizivir (abacavir / lamivudine / zidovudine); Ziagen (abacavir)

For peer review only

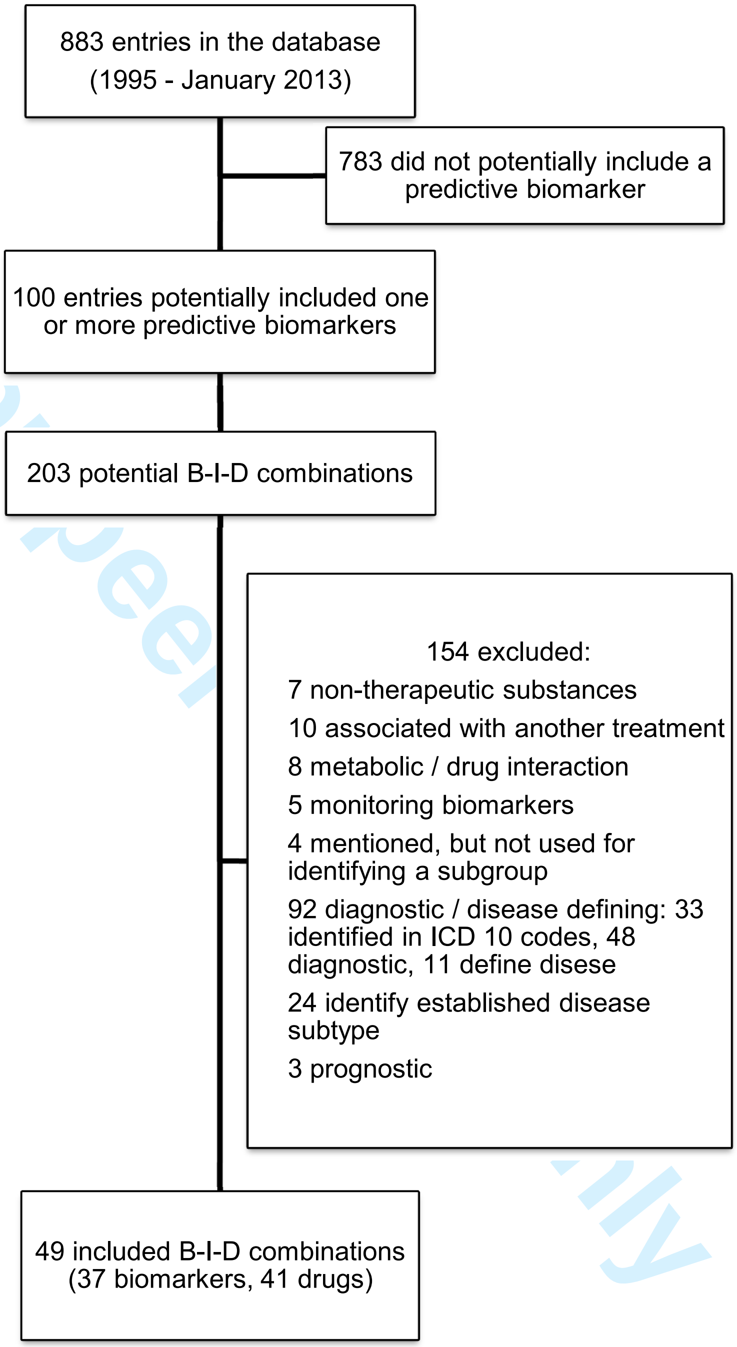


**Figure 1 Flow diagram for the systematic review of predictive biomarkers in EMA licensing**

For peer review only - http://bmjopen.bmj.com/site/about/guidelines.xhtml

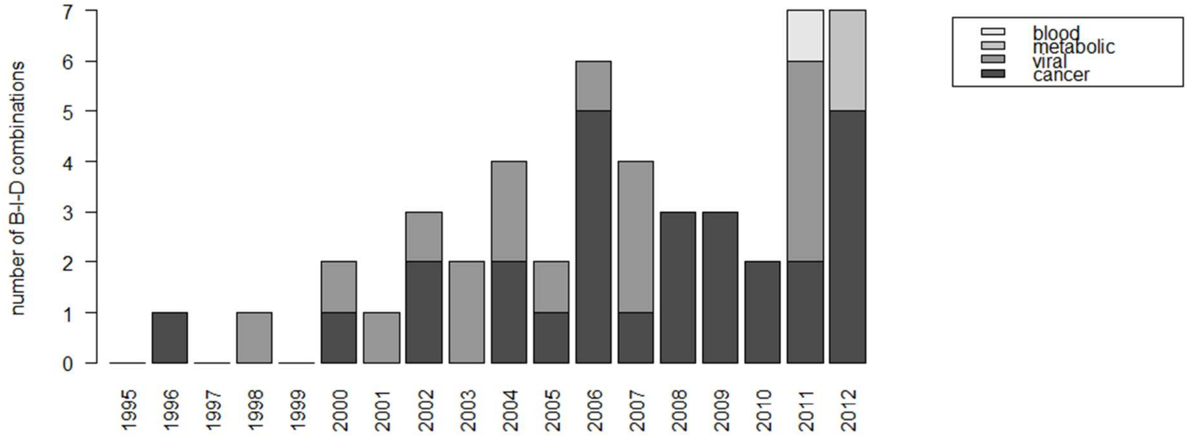


**Figure 2 Number of new B-I-D combinations considered each year by disease area (includes biomarkers added after the drug was initially licensed)**

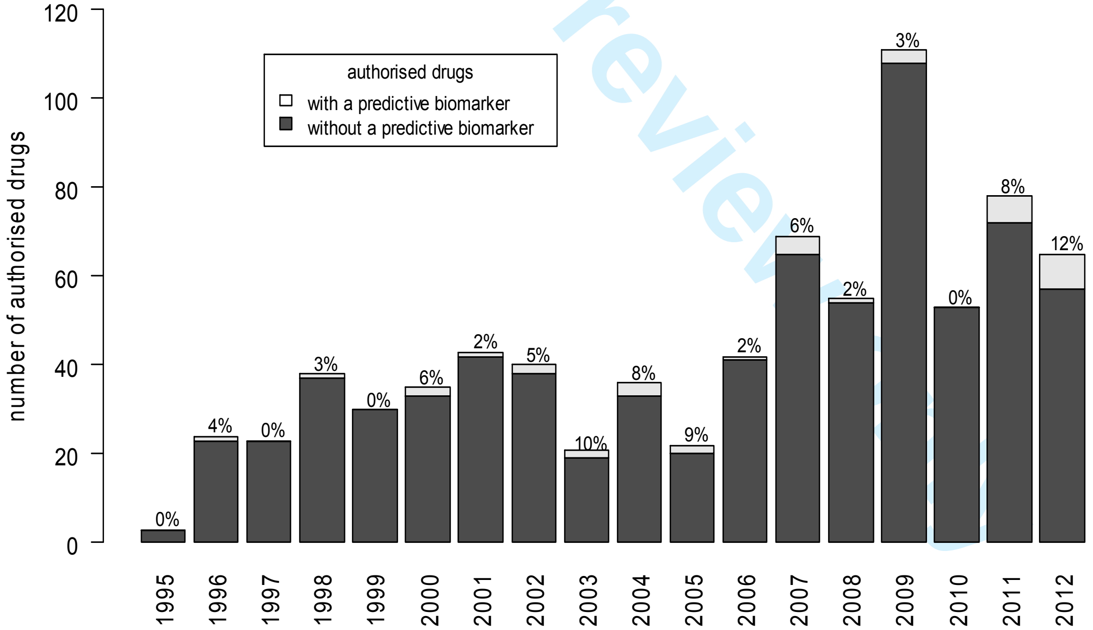


Data for 2012 include 4 generic drugs (capecitabine)

**Figure 3 New drugs authorised each year with and without a predictive biomarker in the indication or contraindication (excludes biomarkers added after the drug was initially licensed)**

For peer review only - http://bmjopen.bmj.com/site/about/guidelines.xhtml

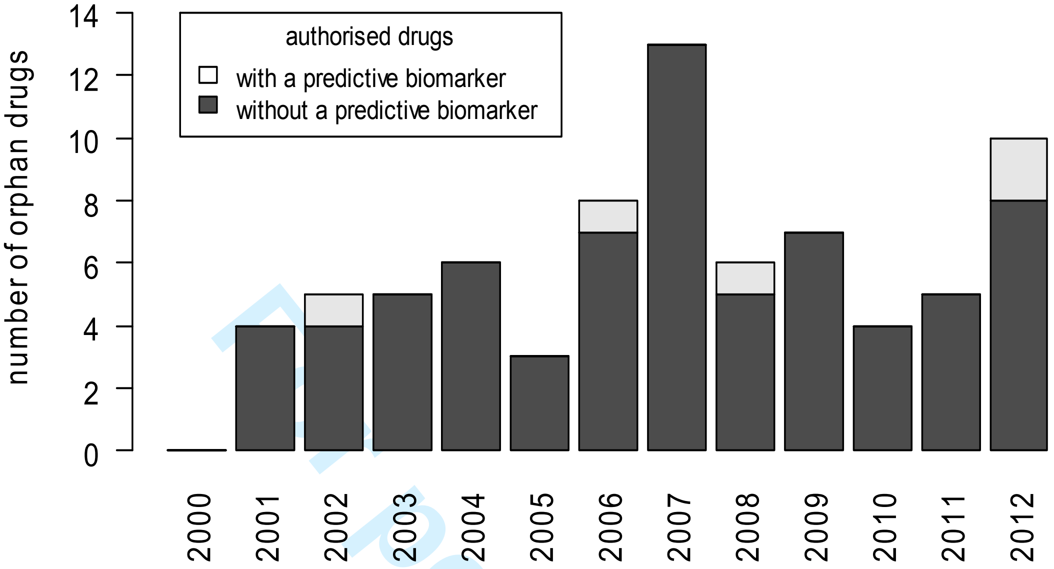


**Figure 4 New drugs orphan authorised each year with and without a predictive biomarker in the indication or contraindication (excludes biomarkers added after the drug was initially licensed)**

For peer review only

**Stratified medicine in European Medicines Agency licensing: a systematic review of predictive biomarkers**

Kinga Malottki*, Mousumi Biswas, Jonathan J. Deeks, Richard D. Riley, Charles Craddock, Philip Johnson, Lucinda Billingham
* corresponding author

Kinga Malottki
Research Fellow
MRC Midland Hub for Trials Methodology Research, University of Birmingham, Birmingham, B15 2TT, UK
k.malottki@bham.ac.uk

Mousumi Biswas
Research Associate
The Discovery Research Programme, School of Social and Community Medicine, University of Bristol, Bristol, BS8 2PS, UK

Jonathan J. Deeks
Professor
Public Health, Epidemiology and Biostatistics, School of Health and Population Sciences, University of Birmingham, Birmingham, B15 2TT, UK
MRC Midland Hub for Trials Methodology Research, University of Birmingham, Birmingham, B15 2TT, UK

Richard D. Riley
Reader
Public Health, Epidemiology and Biostatistics, School of Health and Population Sciences, University of Birmingham, Birmingham, B15 2TT, UK
MRC Midland Hub for Trials Methodology Research, University of Birmingham, Birmingham, B15 2TT, UK

Charles Craddock
Professor
Centre for Clinical Haematology, Queen Elizabeth Hospital, Birmingham, B15 2TH, UK

Philip Johnson
Professor
University of Liverpool & Clatterbridge Cancer Centre NHS Foundation Trust, Liverpool L69 3GA, UK

Lucinda Billingham
Professor
MRC Midland Hub for Trials Methodology Research, University of Birmingham, Birmingham, B15 2TT, UK
Cancer Research UK Clinical Trials Unit, University of Birmingham, Birmingham, B15 2TT, UK

For peer review only

**Abstract**

**Objectives** Stratified medicine is often heralded as the future of clinical practice. Key part of stratified medicine is the use of predictive biomarkers, which identify patient subgroups most likely to benefit (or least likely to experience harm) from an intervention. We investigated how many and what predictive biomarkers are currently included in European Medicines Agency licensing.

**Methods and eligibility criteria** Indications and contraindications of all drugs considered by the EMA and published on their website were screened to identify predictive biomarkers. For all included Biomarker-Indication-Drug (B-I-D) combinations data was collected on: the type of the biomarker, whether it selected a subgroup of patients based on efficacy or toxicity, therapeutic area, marketing status, date of licensing decision, date of inclusion of the biomarker in the indication or contraindication, and on orphan designation.

**Results** 49 B-I-D combinations were identified over 16 years, which included 37 biomarkers and 41 different drugs. All identified biomarkers were molecular. Six drugs (relating to 10 B-I-D combinations) had an orphan designation at the time of licensing. The identified B-I-D combinations were mainly used in cancer and HIV treatment, but also in hepatitis C and three other indications (cystic fibrosis, hyperlipoproteinemia type I, and methemoglobinemia). In 45 B-I-D combinations biomarkers were used as predictive of drug efficacy and in four of drug toxicity. It appeared that there was an increase in the number of B-I-D combinations introduced each year, however the numbers were too small to identify any trends.

**Conclusions** Given the large body of literature documenting research into potential predictive biomarkers and extensive investment into stratified medicine, we identified relatively few predictive biomarkers included in licensing. These were also limited to a small number of clinical areas. This might suggest a need for improvement in methods of translation from laboratory findings to clinical practice.


For peer review only

**Article summary**

**Article focus**

- To identify predictive biomarkers included in European Medicines Agency licensing
- For identified biomarkers, to investigate their type, the clinical areas in which these biomarkers are used and possible trends over time with regard to the number of new predictive biomarkers considered each year

**Key messages**

- 49 Biomarker-Indication-Drug (B-I-D) combinations were identified over 16 years, which included 37 biomarkers and 41 different drugs. There appeared to be an increase in the number of B-I-D combinations introduced each year, however the numbers were too small to identify any trends.
- All identified biomarkers were molecular. They were mainly used in cancer and HIV treatment, but also in hepatitis C and three other indications (cystic fibrosis, hyperlipoproteinemia type I, and methemoglobinemia).

**Strengths and limitations of this study**

- Our research, to our knowledge, provides the first indication of the number and nature of predictive biomarkers included in licensing in Europe using systematic review methodology.
- It is likely that the 49 identified B-I-D combinations do not represent a complete list of predictive biomarkers used in practice, as some could have been considered by national regulatory agencies, particularly for drugs considered before EMA was established in 1995.

For peer review only

**Introduction**

Drugs are rarely effective in all patients and may be associated with serious adverse events.[1] The challenge of stratified medicine is to identify predictive biomarkers that identify patient subgroups (or strata) with a differential therapeutic response to a linked intervention, allowing more appropriate and effective use of interventions to maximise patient benefit and minimise the occurrence of serious adverse events.[2;3] Predictive biomarkers are defined particular to a treatment for a condition, where biomarker values are associated with differential efficacy or toxicity of that treatment.[4-7] The use of predictive biomarkers promises a more appropriate choice of treatment: it can also help to rationalise funding decisions, avoiding costs of futile treatment and of adverse events. However the additional cost of measuring the marker has to be taken into account. Examples of predictive biomarkers include tamoxifen use in breast cancer, which is prescribed to women who are oestrogen receptor positive,[8] and trastuzumab which is prescribed to those with HER2 overexpression in their tumour.[9]

There is a large body of literature documenting research into potential predictive biomarkers,[10;11] and millions of pounds have been invested into stratified medicine, both in industry and through programs from funding bodies such as the Medical Research Council[12] and Cancer Research UK.[13] We aimed to investigate if ~~evaluate the degree to which~~ this ~~investment~~ interest in developing stratified medicines has led to production of biomarker-treatment combinations ready for use in clinical practice. To explore this question, we have undertaken a systematic review of predictive biomarkers reported in licensing decisions of the European Medicines Agency (EMA).

In our review we aimed to find out how many of the indications and contraindications considered by the EMA define a patient population using a predictive biomarker. We were also interested in the disease areas where predictive biomarkers have been used and any trend over time. It has been hypothesised that stratified medicine has not been implemented in practice as much as expected. This paper provides evidence of the impact of stratified medicine research to date and if less than expected, then this will highlight the need to review the underlying reasons and address the problems.

**Methods**

We defined a Biomarker-Indication-Drug (B-I-D) combination as the unit of our analysis, relating to the use of a predictive biomarker with a particular drug for a particular condition or disease.[4-7] For toxicity biomarkers where the a biomarkers of drug toxicity may be used in more than one disease area we grouped these into one B-I-D combination.

All drugs listed on the EMA website in either European Public Assessment Reports or Pending Decisions[14;15] (accessed on the 17th of January 2013) were evaluated, together with their indications and contraindications.

Our inclusion criteria were that the biomarker had to:

For peer review only

(i) be used in the indication and/or contraindication of the drug,
(ii) be associated with a particular treatment,
(iii) identify a subgroup of patients with a particular disease eligible for treatment with the drug.

We excluded biomarkers:

(i) associated with a non-therapeutic substance (for example vaccines),
(ii) not used as predictive, including:
- used for diagnosis, screening or forming part of the disease definition (already established for defining a disease) or established disease subtype,
- prognostic only (associated with outcome regardless of treatment and not predictive of treatment response[16])

(iii) associated with another treatment (for example the biomarker was not associated with the differential efficacy or toxicity of the drug of interest, but another drug given in combination with the drug of interest).

We have reviewed EMA licensing, as in Europe a centralised drug evaluation by the EMA is required for drugs for treatment of a number of conditions, drugs obtained from biotechnology processes and all drugs used for rare conditions (orphan medicines). Companies can also apply for a centralised marketing authorisation of other drugs.[17] Although the EMA does not license biomarkers, it evaluates drugs in groups defined by predictive biomarkers (for example trastuzumab is licensed for use in HER2 overexpressing breast cancer patients).[18] Our approach is likely to give a broad overview of the impact of predictive biomarkers on treatment selection since 1995 (when EMA was established[19]).

We created a database of all drugs in the EMA database including the drug name, licensing status, indication and contraindication. In the first stage of screening all database entries were screened by two independent reviewers (MB and KM) to identify those potentially including a predictive biomarker in the indication or contraindication. If an entry was identified by at least one of the reviewers as potentially relevant, it was included in the second stage of screening.

In the second stage of screening, a list of potential B-I-D combinations was created based on the entries identified in the first stage. The list of potential B-I-D combinations was assessed by two independent reviewers (MB and KM) using full inclusion/exclusion criteria, based on the information in the Summary of Product Characteristics (which sets out the position of the drug obtained in the assessment process and summarises its properties and clinical use together with the clinical trial evidence that was considered by the EMA)[20] , the Scientific Discussion (which discusses the properties and clinical evidence in more detail) and additional information from targeted internet searches and expert advice if necessary. Any disagreements were resolved by discussion.

For the included B-I-D combinations data was collected on: the type of the biomarker used as predictive, whether it selected a subgroup of patients based on efficacy or toxicity, therapeutic area, marketing

For peer review only

status, date of licensing decision, date of inclusion of the biomarker in the indication or contraindication, and on orphan designation (granted to drugs intended for the treatment of a life-threatening or chronically debilitating condition which is either affecting no more than 5 in 10,000 people in the EU or when the revenue is unlikely to cover the investment in drug development[21]). To provide a context for our review, we have also collected data on the total number of drugs licensed each year with and without an orphan designation.

**Results**

Across the 18 year period (1995-2012) we identified 49 B-I-D combinations, including 37 biomarkers and 41 different drugs. The details of the review process are presented in Figure 1~~Figure 1~~. Most of the drugs were authorised, the exceptions being:

- Gemtuzumab ozogamicin (refused)
- Zeldoronic acid (pending)
- Imatinib in the indication for aggressive systemic mastocytosis (withdrawn)
- Amprnavir (withdrawn)
- Nelfinavir (withdrawn)

The number of new B-I-D combinations considered by the EMA each year has increased overall from zero or one per year in the late nineties, to a maximum of 7 in each of 2011 and 2012 as shown in Figure 2~~Figure 2~~. This was however not a steady increase, as the number of B-I-D combinations considered by the EMA showed fluctuation between 2000 and 2006, a decrease between 2006 and 2010, followed by an increase in the number in 2011 and 2012. A predictive biomarker was included in the indication or contraindication at the time when the drug was first licensed for 35 drugs (for one (capecitabine) the date of inclusion of the biomarker was unclear from the documentation, for the remaining drugs the time from the initial licensing decision to the inclusion of a predictive biomarker ranged from one to ten years). The proportion of first licensing decision of all new drugs that included a predictive biomarker increased over time and was close to 10% in 2003, 2004, 2005, 2011 and 2012 (Figure 3).

Six drugs associated with a predictive biomarker had an orphan designation at the time of licensing, however for two it was removed at the end of exclusivity period (details reported in

For peer review only

Table 1Table 1). One of the six drugs (imatinib) was associated with five different predictive biomarkers in five different indications (Figure 4).

The identified predictive biomarkers were all molecular. Thirty-three biomarkers were used to predict treatment efficacy (details reported in

For peer review only

Table 1Table 1) and only four to predict toxicity (Table 2Table 2).

Most of the biomarkers were included in indications and contraindications of cancer treatments (26 B-I-D combinations) and viral diseases, mainly HIV (17 B-I-D combinations). The remaining biomarkers were used to stratify metabolic and blood disorders (cystic fibrosis, hyperlipoproteinemia type I, and methemoglobinemia) and appeared in the last two years (Figure 2Figure 2).

**Discussion**

Stratified medicine is promoted as key to the future of medicine, and is currently one of the most active areas of clinical research. To our knowledge this review provides the first indication of the number and nature of predictive biomarkers included in licensing in Europe based on the drug indications and contraindications on the EMA website. Forty nine B-I-D combinations were identified. All identified biomarkers were molecular. The identified B-I-D combinations were mainly used in cancer and HIV treatment, with only five used in other disease areas.

It is likely that the 49 identified B-I-D combinations from the EMA database do not represent a complete list of the predictive biomarkers used in practice as (some predictive biomarkers could have been considered by national regulatory agencies, particularly for drugs considered before EMA was established in 1995. Also EMA licensing is not compulsory for some disease areas, such as mental health. However a number of drugs with indications in depression of schizophrenia have been considered by the EMA.) Therefore we believe that although our approach might not provide a complete list of all predictive biomarkers used in Europe, relatively few are likely to have been omitted, particularly from recent years.[19] The fact that some of the identified B-I-D combinations included biomarkers introduced to an indication of an already licensed drug suggests that at least to some extent we have captured stratification occurring after the initial licensing of a drug. However the actual extent to which this takes place in clinical practice is difficult to evaluate.

Several types of biomarkers were excluded. We did not include biomarkers used for dose adjustments as they do not directly predict efficacy or toxicity (although inappropriate dose adjustment could limit the treatment efficacy or cause adverse events).[22] We also only investigated biomarkers associated with drug treatments. Other biomarkers may be used in practice with non-drug treatments (for example radiotherapy).

The definition of a predictive biomarker can be difficult to apply, as over time predictive biomarkers may become part of a redefinition of the disease or subtype of disease[23] and be classed as diagnostic tests. In our evaluation we excluded diagnostic biomarkers (for example these included factor IX deficiency, or genetic testing for familial lipoprotein lipase deficiency), and biomarkers used to identify an established subtype of a disease (mainly ST segment elevation and non-ST segment elevation myocardial infarction).

For peer review only

The spectrum of diseases where predictive biomarkers have been successfully developed is relatively narrow. This suggests a possible need for more research in other clinical areas. Also the vast majority of the B-I-D combinations were associated with treatment efficacy and only four with toxicity. As adverse events associated with some treatments could be potentially serious and the possibility to screen out patients at high risk prior to commencing treatment would be beneficial. A proportion of the drugs with an associated predictive biomarker identified in our review had an orphan designation. This seems surprising, as convincing evidence to support the use of a drug in a subgroup of patients with a rare condition might be difficult to obtain, due to the small numbers of patients available to test the hypotheses.

It is difficult to provide accurate estimates of the extent of research into potential predictive biomarkers, however it has been suggested in 2011 that the number of publications on different biomarkers (not only predictive) was in the area of 15 000.[10] Another paper published in 2009, which reviewed genetic markers evaluated as potential predictors of response to treatment, found that 541 different genes were investigated as potential predictive biomarkers in 1 668 papers.[11] It can be reasonably expected that this number largely increased since these papers were published. Our review shows that few predictive biomarkers have been included in licensing relative to th~~e~~is large body of literature documenting numerous potential predictive biomarkers.~~[10;11]~~ Therefore, in spite of the substantial investment in research, the promise of stratified medicine is not yet being realised to a large extent. The reasons for this might include poor translation of findings of laboratory studies into clinical context, or the failure to identify effective predictive biomarkers and treatments. Even though it is becoming easier and cheaper to gather huge sets of genomic data, its interpretation is challenging, which can potentially hinder translational research. Recognising this, initiatives have been undertaken both in the USA (National Institutes of Health and the FDA) and UK (Medical Research Council) to promote the translation of basic research into clinical practice.[12] Also the availability of datasets such as the Cancer Cell Line Encyclopaedia and a similar UK initiative might contribute to the faster progress of stratified medicine.[24;25] The relatively small number of predictive biomarkers identified in licensing might also indicate the need for more sound methodological standards for biomarker discovery and development.[26]

For peer review only

**References**

(1) Xinghua Hu S, Foster T, Kieffaber A. Pharmacogenomics and personalized medicine: mapping future value creation. BioTechniques 2005; 39(4).

(2) Aroon DH, Danielle AvdW, Richard DR, Keith A, Karel GMM, Ewout WS et al. Prognosis research strategy (PROGRESS) 4: Stratified medicine research. BMJ 2013; 346.

(3) Trusheim MR, Berndt ER, Douglas FL. Stratified medicine: strategic and economic implications of combining drugs and clinical biomarkers. Nat Rev Drug Discov 2007; 6(4):287-293.

(4) Mandrekar SJ, Sargent DJ. Predictive biomarker validation in practice: lessons from real trials. Clin Trials 2010.

(5) Sargent DJ, Conley BA, Allegra C, Collette L. Clinical trial designs for predictive marker validation in cancer treatment trials. J Clin Oncol 2005; 23(9):2020-2027.

(6) Simon R. Advances in Clinical Trial Designs for Predictive Biomarker Discovery and Validation. Current Breast Cancer Reports 2009; 1:216-221.

(7) Alymani NA, Smith MD, Williams DJ, Petty RD. Predictive biomarkers for personalised anti-cancer drug use: discovery to clinical implementation. Eur J Cancer 2010; 46(5):869-879.

(8) Jordan VC, Brodie AM. Development and evolution of therapies targeted to the estrogen receptor for the treatment and prevention of breast cancer. Steroids 2007; 72(1):7-25.

(9) Shepard HM, Jin P, Slamon DJ, Pirot Z, Maneval DC. Herceptin. Handb Exp Pharmacol 2008;(181):183-219.

(10) Poste G. Bring on the biomarkers. Nature 2011; 469(7329):156-157.

(11) Holmes MV, Shah T, Vickery C, Smeeth L, Hingorani AD, et al. Fulfilling the promise of personalized medicine? Systematic review and field synopsis of pharmacogenetic studies. PLoS One 2009; 4(12):e7960.

(12) The human genome at ten. Nature 2010; 464(7289):649-650.

(13) Cancer Research UK. Stratified Medicine Programme. http://www.cancerresearchuk.org/science/research/how-we-deliver-our-research/others/by-programme/stratified-medicine-programme/ [ 2011 [cited 2013 Apr. 22];

(14) European public assessment reports. European Medicines Agency [ 2010 [cited 2010 Oct. 19]; Available from: www.ema.europa.eu/ema/index.jsp?curl=/pages/medicines/landing/epar_search.jsp&murl=menus/medicines/medicines.jsp&mid=WC0b01ac058001d124

(15) Pending EC decisions. European Medicines Agency [ 2010 [cited 2010 Oct. 19]; Available from: URL:www.ema.europa.eu/ema/index.jsp?curl=pages/medicines/landing/smop_search.jsp&murl=menus/medicines/medicines.jsp&mid=WC0b01ac058001d127

(16) Simon RM, Paik S, Hayes DF. Use of archived specimens in evaluation of prognostic and predictive biomarkers. J Natl Cancer Inst 2009; 101(21):1446-1452.

For peer review only

(17) European Medicines Agency. Central authorisation of medicines. http://www.ema.europa.eu/ema/index.jsp?curl=pages/about_us/general/general_content_000109.jsp&mid=WC0b01ac0580028a47 [ 2011 [cited 2013 Apr. 22];

(18) European Medicines Agency. Trastuzumab: Summary of product characteristics. http://annonc.oxfordjournals.org/content/12/suppl_1/S57.short [ 2012 [cited 12 A.D. Dec. 20];

(19) European Medicines Agency. How we work. http://www.ema.europa.eu/ema/index.jsp?curl=pages/about_us/general/general_content_000125 jsp&murl=menus/about_us/about_us jsp&mid=WC0b01ac0580028a46 [ 2012 [cited 2012 Feb. 22];

(20) A Giudeline on Summary of Prduct Characteristics. European Medicines Agency [ 2005 [cited 2011 Dec. 15]; Available from: http://ec.europa.eu/health/files/eudralex/vol-2/c/spcguidrev1-oct2005_en.pdf

(21) European Medicines Agency. Orphan Designation. European Medicines Agency [ 2011 [cited 2011 July 28]; Available from: www.ema.europa.eu/ema/index.jsp?curl=pages/regulation/general/general_content_000029.jsp&murl=menus/regulations/regulations.jsp&mid=WC0b01ac05800240ce

(22) Bhathena A, Spear BB. Pharmacogenetics: improving drug and dose selection. Curr Opin Pharmacol 2008; 8(5):639-646.

(23) Bell J. Redefining disease. Harveian Oration 2010.

(24) Barretina J, Caponigro G, Stransky N, Venkatesan K, Margolin AA, et al. The Cancer Cell Line Encyclopedia enables predictive modelling of anticancer drug sensitivity. Nature 2012; 483(7391):603-607.

(25) Garnett MJ, Edelman EJ, Heidorn SJ, Greenman CD, Dastur A, et al. Systematic identification of genomic markers of drug sensitivity in cancer cells. Nature 2012; 483(7391):570-575.

(26) Janes H, Pepe MS, Bossuyt PM, Barlow WE. Measuring the performance of markers for guiding treatment decisions. Ann Intern Med 2011; 154(4):253-259.

For peer review only

### Licence

The Corresponding Author has the right to grant on behalf of all authors and does grant on behalf of all authors, an exclusive licence (or non exclusive for government employees) on a worldwide basis to the BMJ Publishing Group Ltd to permit this article (if accepted) to be published in BMJ editions and any other BMJPGL products and sublicences such use and exploit all subsidiary rights, as set out in our licence.

### Competing interests

All authors declare that they have no financial or non-financial interests that may be relevant to the submitted work.

### Author Contributions

KM, JD, RR and LB designed the review. KM and MB carried out the review. Where needed CC and PJ provided clinical advice. All authors contributed to the interpretation of the results, commented on drafts and accepted the final version of this paper.

The manuscript is an honest, accurate, and transparent account of the study being reported; no important aspects of the study have been omitted; any discrepancies from the study as planned have been explained.

### Data sharing

Data sharing: full dataset available from the corresponding author.

### Ethical Approval

No ethical approval was required, as the study did not involve collection of patient data

### Study funding

This work was funded by the MRC Midlands Hub for Trials Methodology Research at the University of Birmingham (Medical Research Council Grant ID G0800808). The funders had no role in study design, data collection and analysis, decision to publish, or preparation of the manuscript.

For peer review only

**Table 1 Biomarkers predictive of efficacy identified in the review of EMA licensing**

| Biomarker | Indication | Drug |
|---|---|---|
| **ALK gene rearrangement** | Carcinoma, Non-Small-Cell Lung | Crizotinib (Xalkori) |
| **BRAF V600 mutation** | Melanoma | Vemurafenib (Zelboraf) |
| **CCR5 tropism** | HIV Infections | Maraviroc (Celsentri) |
| **CD-33 expression*** | Leukemia, Myeloid, Acute | gemtuzumab ozogamicin (Mylotarg)[‡] |
| **EGFR expression** | Colorectal Neoplasms | Cetuximab (Erbitux) |
| **EGFR expression** | Carcinoma, Non-Small-Cell Lung | Erlotinib (Tarceva) |
| **EGFR mutation** | Carcinoma, Non-Small-Cell Lung | Erlotinib (Tarceva) |
| **EGFR mutation** | Carcinoma, Non-Small-Cell Lung | Gefitinib (Iressa) |
| **EpCAM expression** | CancerAscites | Catumaxomab (Removab) |
| **FIP1L1-PDGFR rearrangement** | Hypereosinophilic Syndrome | Imatinib (Glivec)[#] |
| **G551D mutation in the CFTR gene** | Cystic Fibrosis | Ivacaftor (Kalydeco)[‡] |
| **genotype 1 HCV** | Hepatitis C, Chronic | Boceprevir (Victrelis) |
| **genotype 1 HCV** | Hepatitis C | Telaprevir (Incivo) |
| **HER2 expression** | Breast Neoplasms | Lapatinib (Tyverb) |
| **HER2 expression** | Breast Neoplasms | Trastuzumab (Herceptin) |
| **HER2 expression** | Stomach Neoplasms | Trastuzumab (Herceptin) |
| **HER2 expression** | Breast Neoplasms | Everolimus (Afinitor) |
| **HER2 expression **** | Breast Neoplasms | pertuzumab (Perjeta) |
| **Hormone dependency** | Prostatic Neoplasms | Degarelix (Firmagon) |
| **Hormone receptor expression**** | Breast Neoplasms | zoledronic acid (Zometa) |
| **Hormone receptor expression** | Breast Neoplasms | Everolimus (Afinitor) |
| **Kit (CD 117) expression** | Gastrointestinal Stromal Tumors | Imatinib (Glivec)[#] |
| **Kit (D816V) mutation***** | Aggressive Systemic Mastocytosis | Imatinib (Glivec)[#] |
| **KRAS mutation** | Colorectal Neoplasms | Cetuximab (Erbitux) |
| **KRAS mutation** | Colorectal Neoplasms | Panitumumab (Vectibix) |
| **LPL protein detectable** | Hyperlipoproteinemia Type I | alipogene tiparvovec (Glybera)[‡] |
| **oestrogen receptor expression** | Breast Neoplasms | Fulvestrant (Faslodex) |
| **oestrogen receptor expression** | Breast Neoplasms | Toremifene (Fareston) |
| **PDGFR gene rearrangements** | Myelodysplastic-Myeloproliferative Diseases | Imatinib (Glivec)[#] |
| **Philadelphia chromosome** | Precursor Cell Lymphoblastic Leukemia-Lymphoma | Dasatinib (Sprycel)[‡] |
| **Philadelphia chromosome** | Precursor Cell Lymphoblastic Leukemia-Lymphoma | Imatinib (Glivec)[#] |
| **t(15;17) translocation** | Leukemia, Promyelocytic, Acute | arsenic trioxide (Trisenox)[#] |
| **viral resistance mutations***** | HIV Infections | Amprenavir (Agenerase) |
| **viral resistance mutations** | HIV Infections | atazanavir sulphate (Reyataz) |
| **viral resistance mutations** | HIV Infections | Darunavir (Prezista) |
| **viral resistance mutations** | HIV Infections | efavirenz / emtricitabine / tenofovir disoproxil (Atripla) |
| **viral resistance mutations** | HIV Infections | Emtricitabine (Emtriva) |
| **viral resistance mutations** | HIV Infections | emtricitabine / rilpivirine / tenofovir disoproxil (Eviplera) |
| **viral resistance mutations** | HIV Infections | Enfuvirtide (Fuzeon) |

For peer review only

| | | |
|---|---|---|
| **viral resistance mutations** | HIV Infections | fosamprenavir calcium (Telzir) |
| **viral resistance mutations** | HIV Infections | lopinavir / ritonavir (Kaletra) |
| **viral resistance mutations** *** | HIV Infections | Nelfinavir (Viracept) |
| **viral resistance mutations** | HIV Infections | rilpivirine hydrochloride (Edurant) |
| **viral resistance mutations** | HIV Infections | tenofovir disoproxil fumarate (Viread) |
| **viral resistance mutations** | HIV Infections | Tipranavir (Aptivus) |

* refused **pending ***withdrawn
‡ drug designated an orphan medicine, # orphan designation has been removed at the end of exclusivity period

**Table 2 Biomarkers predictive of toxicity identified in the review of EMA licensing**

| Biomarker | Indication | Drug |
|---|---|---|
| **DPD deficiency** | Colorectal Neoplasms<br>Colonic Neoplasms<br>Stomach Neoplasms<br>Breast Neoplasms | Capecitabine (Xeloda and generic drugs: Capecitabine Accord; Capecitabine Krka; Capecitabine Medac; Capecitabine Teva) |
| **DPD deficiency** | Stomach Neoplasms | tegafur / gimeracil / oteracil (Teysuno) |
| **HLA-B*5701 allele** | HIV Infections | Abacavir (Kivexa; Trizivir; Ziagen)* |
| **NADPH reductase deficiency** | Methemoglobinemia | Methylthioninium chloride (Methylthioninium chloride Proveblue) |

* HLA-B*5701 allele is predictive of hypersensitivity to abacavir, which is present in three three drugs: Kivexa (abacavir / lamivudine); Trizivir (abacavir / lamivudine / zidovudine); Ziagen (abacavir)

For peer review only

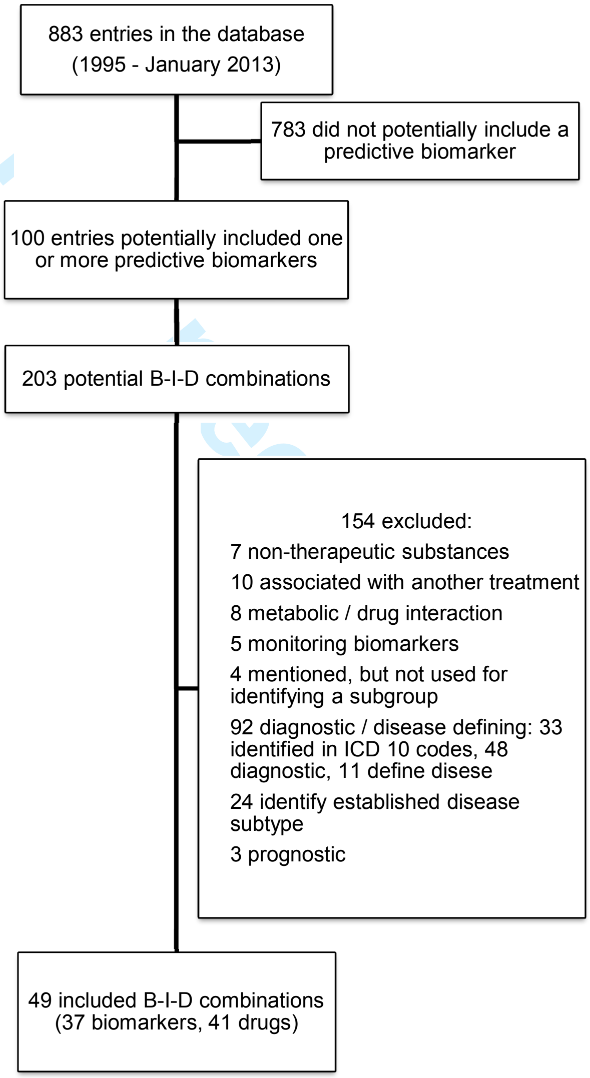


**Figure 1 Flow diagram for the systematic review of predictive biomarkeres in EMA ~~licensig~~licensing**

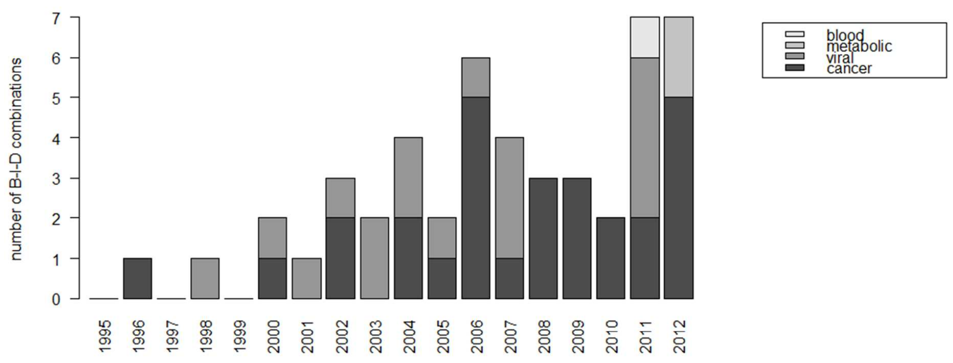


**Figure 2 Number of new B-I-D combinations considered each year by disease area** **(includes biomarkers added after the drug was initially licensed)**

**Formatted:** Font: (Default) Arial

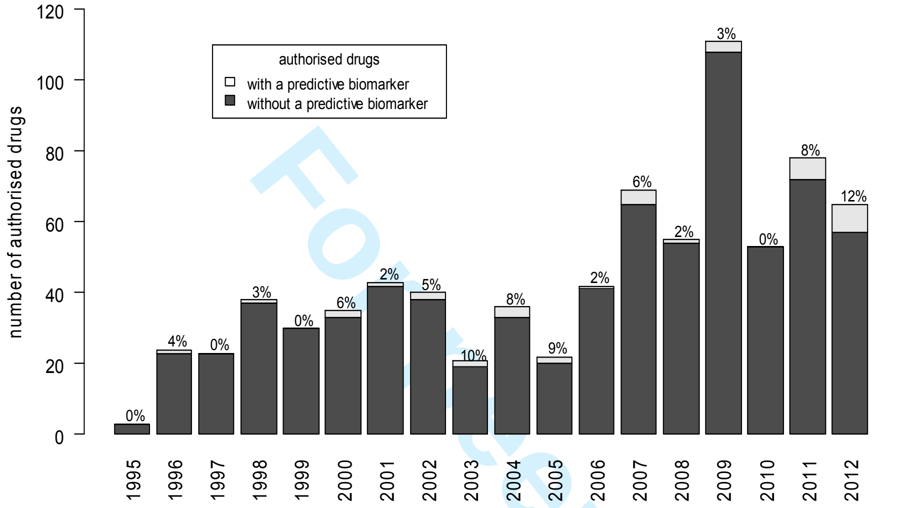


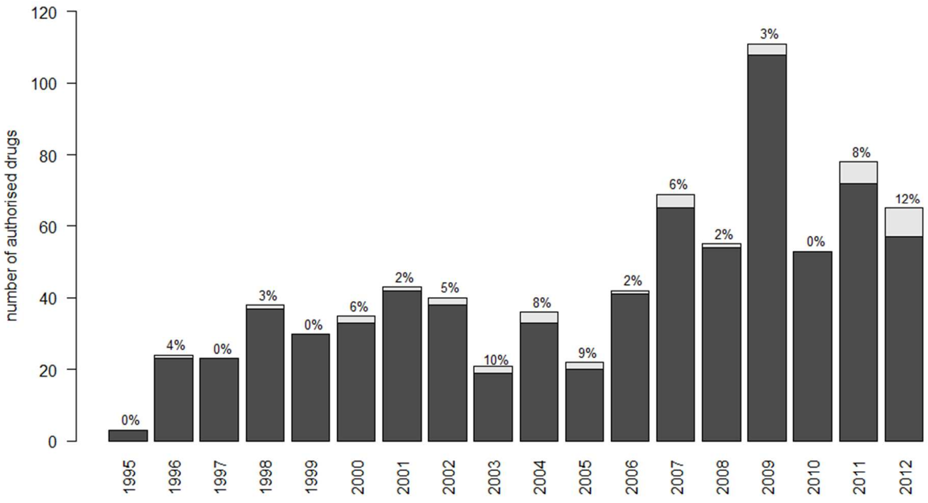


Data for 2012 include 4 generic drugs (capecitabine)

**Figure 3 New drugs authorised each year with and without a predictive biomarker in the indication or contraindication (excludes biomarkers added after the drug was initially licensed)**

Formatted: Font: (Default) Arial

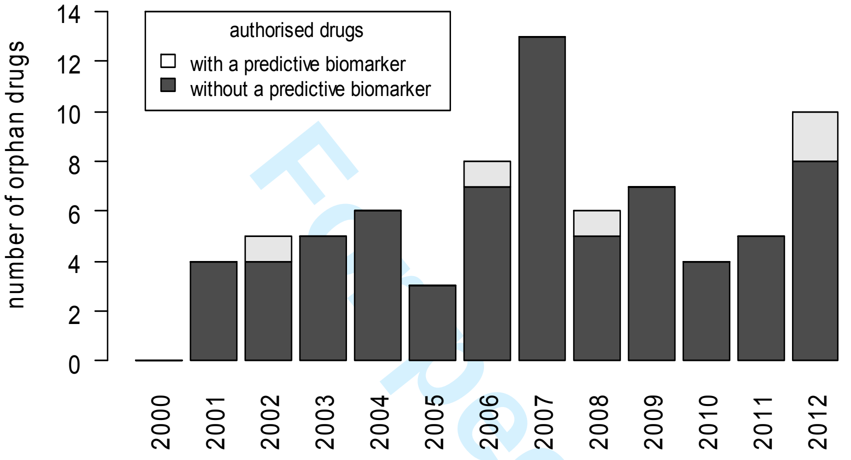


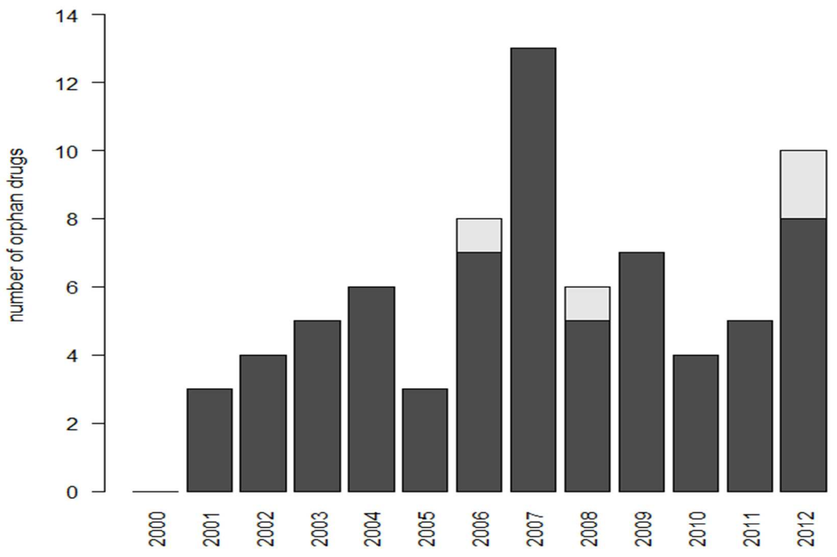


**Figure 4 New drugs orphan authorised each year with and without a predictive biomarker in the indication or contraindication (excludes biomarkers added after the drug was initially licensed)**

For peer review only

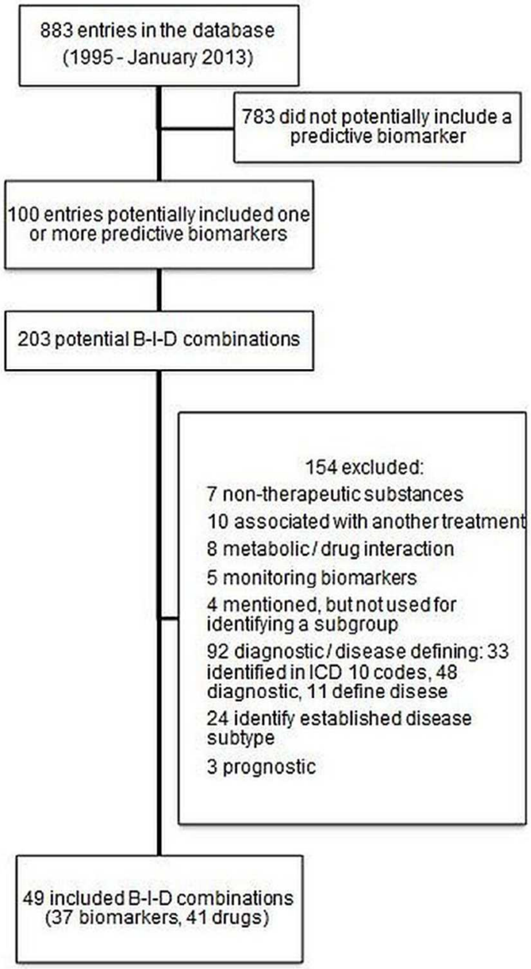


Figure 1 Flow diagram for the systematic review of predictive biomarkers in EMA licensing

67x90mm (300 x 300 DPI)

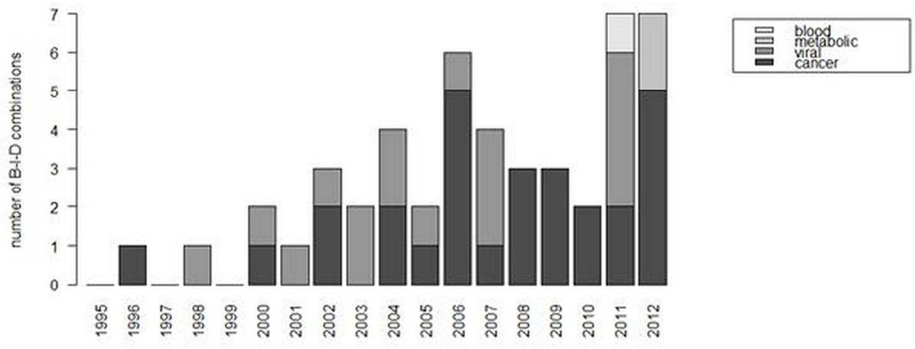


Figure 2 Number of new B-I-D combinations considered each year by disease area (includes biomarkers added after the drug was initially licensed)

151x90mm (300 x 300 DPI)

review only

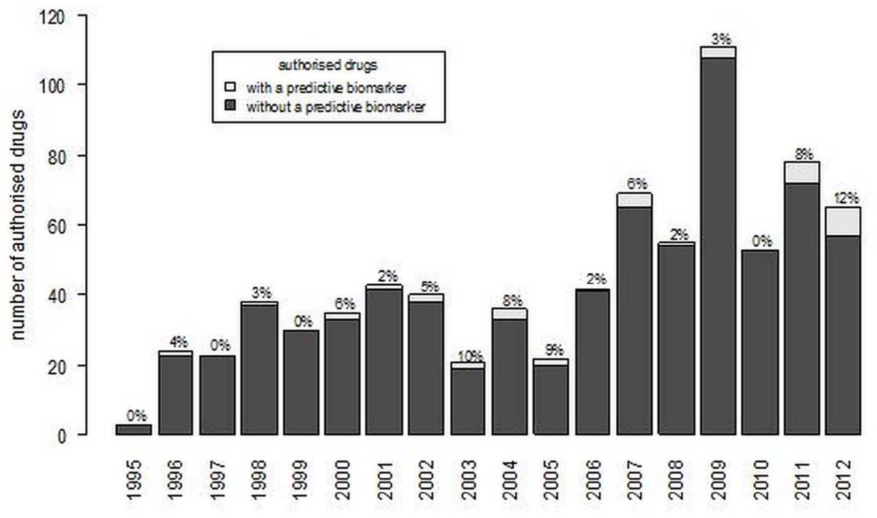


Data for 2012 include 4 generic drugs (capecitabine)

**Figure 3 New drugs authorised each year with and without a predictive biomarker in the indication or contraindication (excludes biomarkers added after the drug was initially licensed)**

123x90mm (300 x 300 DPI)

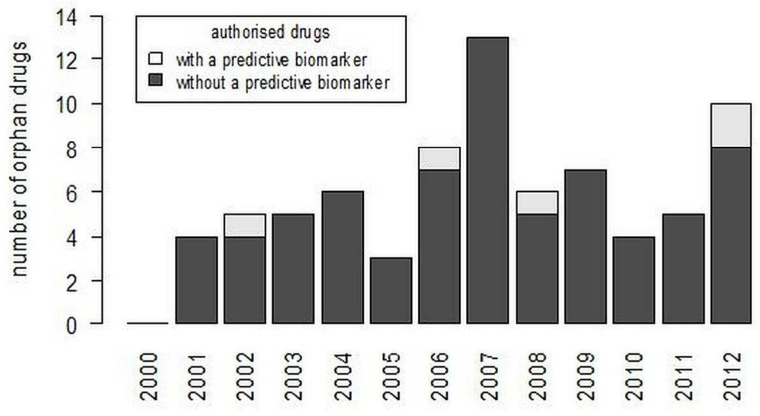


**Figure 4 New drugs orphan authorised each year with and without a predictive biomarker in the indication or contraindication (excludes biomarkers added after the drug was initially licensed)**

148x90mm (300 x 300 DPI)

review only

# PRISMA 2009 Checklist

| Section/topic | # | Checklist item | Reported on page # |
|---|---|---|---|
| **TITLE** | | | |
| Title | 1 | Identify the report as a systematic review, meta-analysis, or both. | 1 |
| **ABSTRACT** | | | |
| Structured summary | 2 | Provide a structured summary including, as applicable: background; objectives; data sources; study eligibility criteria, participants, and interventions; study appraisal and synthesis methods; results; limitations; conclusions and implications of key findings; systematic review registration number. | 2 |
| **INTRODUCTION** | | | |
| Rationale | 3 | Describe the rationale for the review in the context of what is already known. | 3 |
| Objectives | 4 | Provide an explicit statement of questions being addressed with reference to participants, interventions, comparisons, outcomes, and study design (PICOS). | 3 |
| **METHODS** | | | |
| Protocol and registration | 5 | Indicate if a review protocol exists, if and where it can be accessed (e.g., Web address), and, if available, provide registration information including registration number. | 2 |
| Eligibility criteria | 6 | Specify study characteristics (e.g., PICOS, length of follow-up) and report characteristics (e.g., years considered, language, publication status) used as criteria for eligibility, giving rationale. | 3-4 |
| Information sources | 7 | Describe all information sources (e.g., databases with dates of coverage, contact with study authors to identify additional studies) in the search and date last searched. | 3-4 |
| Search | 8 | Present full electronic search strategy for at least one database, including any limits used, such that it could be repeated. | 3 |
| Study selection | 9 | State the process for selecting studies (i.e., screening, eligibility, included in systematic review, and, if applicable, included in the meta-analysis). | 4 |
| Data collection process | 10 | Describe method of data extraction from reports (e.g., piloted forms, independently, in duplicate) and any processes for obtaining and confirming data from investigators. | 4-5 |
| Data items | 11 | List and define all variables for which data were sought (e.g., PICOS, funding sources) and any assumptions and simplifications made. | 4-5 |
| Risk of bias in individual studies | 12 | Describe methods used for assessing risk of bias of individual studies (including specification of whether this was done at the study or outcome level), and how this information is to be used in any data synthesis. | Not applicable |
| Summary measures | 13 | State the principal summary measures (e.g., risk ratio, difference in means). | Not applicable |

For peer review only

For peer review only - http://bmjopen.bmj.com/site/about/guidelines.xhtml

# PRISMA 2009 Checklist

| | | | |
|---|---|---|---|
| Synthesis of results | 14 | Describe the methods of handling data and combining results of studies, if done, including measures of consistency (e.g., $I^2$) for each meta-analysis. | Not applicable |



| Section/topic | # | Checklist item | Reported on page # |
|---|---|---|---|
| Risk of bias across studies | 15 | Specify any assessment of risk of bias that may affect the cumulative evidence (e.g., publication bias, selective reporting within studies). | Not applicable |
| Additional analyses | 16 | Describe methods of additional analyses (e.g., sensitivity or subgroup analyses, meta-regression), if done, indicating which were pre-specified. | Not applicable |
| **RESULTS** | | | |
| Study selection | 17 | Give numbers of studies screened, assessed for eligibility, and included in the review, with reasons for exclusions at each stage, ideally with a flow diagram. | 13 |
| Study characteristics | 18 | For each study, present characteristics for which data were extracted (e.g., study size, PICOS, follow-up period) and provide the citations. | Not applicable |
| Risk of bias within studies | 19 | Present data on risk of bias of each study and, if available, any outcome level assessment (see item 12). | Not applicable |
| Results of individual studies | 20 | For all outcomes considered (benefits or harms), present, for each study: (a) simple summary data for each intervention group (b) effect estimates and confidence intervals, ideally with a forest plot. | Not applicable |
| Synthesis of results | 21 | Present results of each meta-analysis done, including confidence intervals and measures of consistency. | Not applicable |
| Risk of bias across studies | 22 | Present results of any assessment of risk of bias across studies (see Item 15). | Not applicable |
| Additional analysis | 23 | Give results of additional analyses, if done (e.g., sensitivity or subgroup analyses, meta-regression [see Item 16]). | Not applicable |
| **DISCUSSION** | | | |
| Summary of evidence | 24 | Summarize the main findings including the strength of evidence for each main outcome; consider their relevance to key groups (e.g., healthcare providers, users, and policy makers). | 6 |
| Limitations | 25 | Discuss limitations at study and outcome level (e.g., risk of bias), and at review-level (e.g., incomplete retrieval of identified research, reporting bias). | 6 |
| Conclusions | 26 | Provide a general interpretation of the results in the context of other evidence, and implications for future research. | 6-7 |
| **FUNDING** | | | |

For peer review only

For peer review only - http://bmjopen.bmj.com/site/about/guidelines.xhtml

# PRISMA 2009 Checklist

| | | | |
|---|---|---|---|
| Funding | 27 | Describe sources of funding for the systematic review and other support (e.g., supply of data); role of funders for the systematic review. | 10 |

*From:* Moher D, Liberati A, Tetzlaff J, Altman DG, The PRISMA Group (2009). Preferred Reporting Items for Systematic Reviews and Meta-Analyses: The PRISMA Statement. PLoS Med 6(6): e1000097. doi:10.1371/journal.pmed1000097

For more information, visit: **www.prisma-statement.org**.



For peer review only

For peer review only - http://bmjopen.bmj.com/site/about/guidelines.xhtml
